# A chalcone-related small molecule that induces methuosis, a novel form of non-apoptotic cell death, in glioblastoma cells

**DOI:** 10.1186/1476-4598-10-69

**Published:** 2011-06-06

**Authors:** Jean H Overmeyer, Ashley M Young, Haymanti Bhanot, William A Maltese

**Affiliations:** 1 Department of Biochemistry and Cancer Biology, University of Toledo College of Medicine, Toledo, Ohio, USA

## Abstract

**Background:**

Methuosis is a unique form of non-apoptotic cell death triggered by alterations in the trafficking of clathrin-independent endosomes, ultimately leading to extreme vacuolization and rupture of the cell.

**Results:**

Here we describe a novel chalcone-like molecule, 3-(2-**m**ethyl-1H- **i**ndol-3-yl)-1-(4-**p**yridinyl)-2-**p**ropen-1-one (MIPP) that induces cell death with the hallmarks of methuosis. MIPP causes rapid accumulation of vacuoles derived from macropinosomes, based on time-lapse microscopy and labeling with extracellular fluid phase tracers. Vacuolization can be blocked by the cholesterol-interacting compound, filipin, consistent with the origin of the vacuoles from non-clathrin endocytic compartments. Although the vacuoles rapidly acquire some characteristics of late endosomes (Rab7, LAMP1), they remain distinct from lysosomal and autophagosomal compartments, suggestive of a block at the late endosome/lysosome boundary. MIPP appears to target steps in the endosomal trafficking pathway involving Rab5 and Rab7, as evidenced by changes in the activation states of these GTPases. These effects are specific, as other GTPases (Rac1, Arf6) are unaffected by the compound. Cells treated with MIPP lose viability within 2-3 days, but their nuclei show no evidence of apoptotic changes. Inhibition of caspase activity does not protect the cells, consistent with a non-apoptotic death mechanism. U251 glioblastoma cells selected for temozolomide resistance showed sensitivity to MIPP-induced methuosis that was comparable to the parental cell line.

**Conclusions:**

MIPP might serve as a prototype for new drugs that could be used to induce non-apoptotic death in cancers that have become refractory to agents that work through DNA damage and apoptotic mechanisms.

## Background

Cancer cells typically harbor mutations in tumor suppressor genes that control programmed cell death, rendering them relatively insensitive to apoptosis. Moreover, many tumors that initially respond to treatment with standard chemotherapeutic drugs eventually develop multi-drug resistance due to increases in drug efflux mechanisms or DNA repair capacity [1,2]. These challenges have stimulated interest in identifying alternative cell death pathways that might be used to kill tumor cells that have ceased to respond to drugs that depend on induction of apoptotic mechanisms.

Several different forms of non-apoptotic cell death have been described, based on specific morphological or molecular criteria [3,4]. These include death associated with accumulation of autophagosomes [5-7], as well as several types of caspase-independent cell death that may represent specialized forms of necrosis; *e.g*., oncosis [8-10], necroptosis [11,12] and paraptosis [13,14]. More than a decade ago Chi *et al*. [15] reported a unique type of non-apoptotic cell death that can be induced in glioblastoma and gastric carcinoma cells by constitutive stimulation of Ras signaling pathways. We have shown that this form of cell death is distinct from other kinds of non-apoptotic death noted above [16]. It involves stimulation of macropinocytosis (cell drinking), combined with defects in clathrin-independent endocytic vesicle trafficking, ultimately resulting in accumulation of large vacuoles that disrupt cellular membrane integrity. We have termed this form of cell death 'methuosis', from the Greek *methuo*, to drink to intoxication. Mechanistically, the effects of Ras overexpression are related to activation of Rac1 and inactivation of Arf6, two GTPases implicated in macropinocytosis and endosome recycling, respectively [17].

Since our initial description of Ras-induced methuosis, others have reported similar forms of cell death associated with accumulation of macropinosome-derived vacuoles in various contexts, including: treatment of TrkA-positive medulloblastoma cells with nerve growth factor [18], exposure of neurons to methamphetamine [19], and treatment of prostate cancer cells with a nucleolin-binding oligonucleotide aptamer, AS1411 [20]. These studies lend credence to the idea that methuosis may represent a non-apoptotic cell death mechanism of some general importance. However, the potential for exploiting this non-conventional cell death pathway to kill cancer cells that are refractory to apoptosis will depend on the identification of molecules with drug-like properties that can induce methuosis. Toward this end, we now describe a chalcone-related compound that can induce cell death with the hallmarks of methuosis in both temozolomide-resistant and non-resistant glioblastoma cells. This compound may serve as a prototype for a new class of therapeutic agents that could be used to treat tumors that are resistant to conventional drugs.

## Results

### Small Molecules that Induce Cytoplasmic Vacuolization

**Figure 1:**
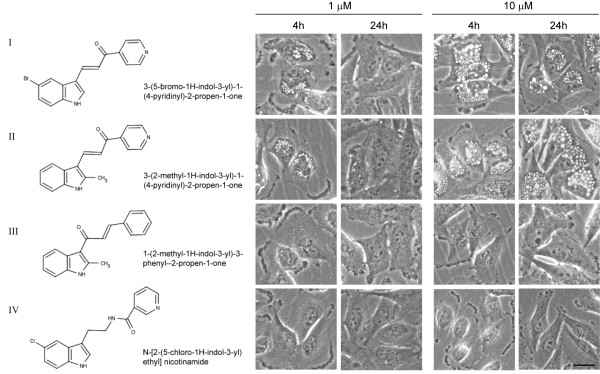
**Compounds, I and II, induce extreme cytoplasmic vacuolization in U251 glioblastoma cells**. Cells were seeded at 500,000 cells in 60 mm dishes. The day after plating, cells were treated with either 1 μM or 10 μM of the indicated compounds. Phase-contrast images were taken 4 h and 24 h after addition of the drugs. The scale bar is 10 microns and applies to all of the panels. The appearance of the cells treated with compounds III and IV was identical to control cultures treated with an equivalent volume of DMSO (not shown). Compound II was selected for further study and was designated MIPP.

We began our search for drug-like compounds that might induce methuosis by surveying the literature for reports of molecules that cause forms of cell vacuolization resembling that induced by overexpression of activated H-Ras in glioma and other cancer cell lines [15-17]. We noted a report from Kirchhausen and colleagues [21] in which they described a group of 16 vacuole-inducing compounds (vacuolins) identified in an image-based phenotypic screen of the Chembridge Diverset E library. Thirteen of the compounds had similar triazine-based core structures, and the most potent of these was named vacuolin-1. The vacuoles induced by vacuolin-1 were found to originate from inappropriate homotypic fusion and swelling of endosomes and lysosomes, but vacuolin-1 was reported not to interfere with cell growth or viability [21]. In surveying the remaining uncharacterized vacuole-inducing compounds mentioned in this study, one unique molecule, 3-(5-bromo-1H-indol-3-yl)-1-(4-pyridinyl)-2-propen-1-one (Figure 1, compound I), captured our attention because of its resemblance to chalcones, naturally occurring flavonoid precursors with a 1,3-diphenyl- 2-propen-1-one framework [22]. Synthetic derivatives built on the chalcone framework have recently been shown to exhibit anti-cancer activity [22-24].

We found that compound I caused a striking accumulation of numerous phase-lucent cytoplasmic vacuoles within 4 h when applied to U251 glioblastoma cells (Figure 1). When added at a concentration of 1 μM, the morphological effects of compound I were transient, with most of the vacuoles dissipating by 24 h (Figure 1). However, at 10 μM, the morphological effects of compound I persisted for 24 h and beyond. A search of the broader 700,000 compound Chembridge collection yielded several additional compounds with >75% similarity to compound I. Of these, 3-(2-methyl-1H- indol-3-yl)-1-(4-pyridinyl)- 2-propen-1-one (Figure 1, compound II), behaved similarly to compound I when tested at 1 μM, but induced vacuoles that were larger and more numerous than those induced by compound I when tested at a concentration of 10 μM (Figure 1). Closely related compounds with similar or identical indole ring structures, but with variations in the second aryl ring (compound III) or the enone linker (compound IV), showed no vacuole-inducing activity (Figure 1). This suggested that the effects of compounds I and II were probably due to their interactions with specific intracellular targets, rather than non-specific effects on cellular membranes or intracellular pH. Compound III had no vacuole-inducing activity despite the fact that it shared the characteristics of a Michael acceptor with compounds I and II. This makes it unlikely that the activities of compounds I and II were due to general covalent protein modification by Michael addition [25,26]. Based on these initial observations, compound II was selected for further study as a potential small molecule inducer of methuosis. Hereafter it will be referred to by the acronym MIPP: *i.e*., 3-(2-**m**ethyl-1H **i**ndol-3-yl)-1-(4-**p**yridinyl)-2-**p**ropen-1-one.

### The Origin of the Vacuoles Induced by MIPP is Consistent with Methuosis

**Figure 2:**
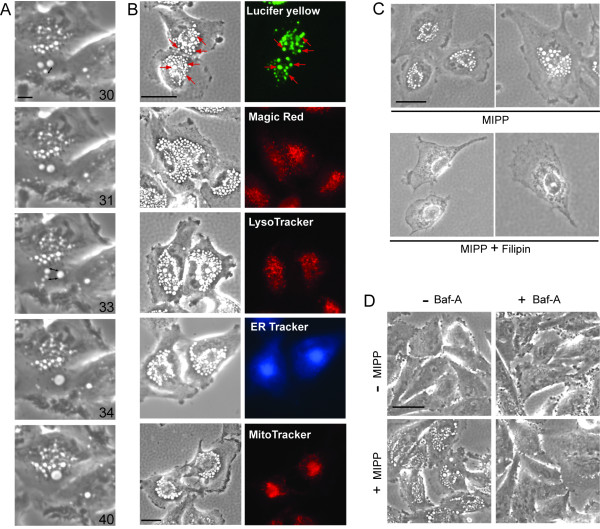
**Vacuoles induced by MIPP are derived from macropinosomes that undergo progressive fusion events and accumulate at a pre-lysosomal stage**. A) Time-lapse phase-contrast microscopy of U251 cells treated with MIPP. Images were captured at 30 sec intervals during the period between 13-80 min after the addition of 10 μM MIPP, and the images were assembled into a movie, which is available as Movie 1 (Additional file 1). The panels show sequential snapshots from the movie, with the elapsed time after addition of MIPP (min) indicated. Newly formed macropinosomes can be seen fusing with each other to form larger vacuoles. The small two-headed arrows point to vesicles that have fused in the subsequent frame. B) U251 cells were treated with 10 μM MIPP for 4 h, then incubated with the indicated fluorescent tracer or organelle marker, as described in Materials and Methods. The same field of cells is depicted in the matching phase-contrast and fluorescent images. In the top panel, the arrows indicate some of the specific vacuoles that have incorporated the Lucifer yellow. C) Cells were pretreated for 30 min with 12 μg/ml filipin or an equivalent volume of vehicle (DMSO), then 10 μM MIPP was added to the cultures. Phase-contrast images were acquired 100 min after the addition of the MIPP. D) Bafilomycin A1 (Baf-A) blocks the induction of vacuoles by MIPP. Cells were pretreated for 1 h in the presence (+) or absence (-) of 100 nM Baf A prior to addition of MIPP (+MIPP) or DMSO (-MIPP). Phase-contrast images were taken 1 h after addition of MIPP. The scale bars in all of the images represent 10 microns.

We first wished to determine if the vacuoles induced by MIPP were derived from macropinosomes, since this is a hallmark of methuosis. Macropinocytosis is a form of clathrin-independent endocytosis wherein intracellular vesicles are initially generated from projections of the plasma membrane termed ruffles or lamellipodia, which surround and trap extracellular fluid [27]. Time lapse phase-contrast microscopy covering the period between 13-80 min after addition of MIPP revealed waves of macropinocytotic vesicles entering the U251 cells from regions of active membrane ruffling. The nascent vesicles could be seen coalescing with each other to form progressively larger vacuoles within the cytoplasm (Figure 2A and Additional file 1, Movie 1). Time lapse studies performed after the first 95 min revealed a decline in the initial burst of macropinocytotic activity, although the vesicles already formed within the cell continued to enlarge by undergoing occasional fusion events (Additional file 2, Movie 2).

One of the features of methuosis, as previously defined in glioblastoma cells expressing activated H-Ras, is the incorporation of fluid-phase tracers like Lucifer yellow (LY) into large vacuoles that eventually fill the cytoplasm and disrupt the cells [16]. Therefore, to confirm that the MIPP-induced vacuoles observed by phase-contrast microscopy were indeed derived from macropinosomes, U251 cells were incubated with LY during the first 4 h after addition of the compound. As shown in Figure 2B, LY was incorporated into most of the phase-lucent vacuoles.

Macropinocytosis, which is a clathrin-independent form of endocytosis, depends on the integrity of cholesterol-rich membrane microdomains [28]. There is evidence that binding and sequestration of cholesterol by treatment of cells with filipin selectively impairs clathrin-independent endocytosis, while clathrin-dependent receptor mediated endocytosis is unaffected [29]. Therefore, to establish that the MIPP-induced vacuoles were indeed derived from clathrin-independent compartments, U251 cells were pre-incubated with or without filipin for 30 min prior to adding MIPP. As shown in Figure 2C, cells treated with MIPP in the absence of filipin formed numerous vacuoles within the first 100 min after addition of the compound. In contrast, the cells treated with filipin failed to show the typical morphological response to MIPP. Thus, the majority of the vacuoles induced by MIPP appear to originate from clathrin-independent macropinosomes, consistent with the mechanism of methuosis.

### Relationship of MIPP-Induced Vacuoles to Other Subcellular Compartments

In Ras-induced methuosis, the accumulated vacuoles eventually acquire some characteristics of late endosomes, but remain separate from the endoplasmic reticulum and the lysosomal or autophagosomal degradative compartments [16]. The vacuoles induced by MIPP clearly fit this profile. Live cell imaging of the phase-lucent vacuoles induced by MIPP showed no overlap with compartments labeled with LysoTracker Red, which identifies lysosomes based on their acidic pH, or Magic Red (RR), a cell permeable substrate that fluoresces when cleaved by the lysosomal protease, cathepsin B (Figure 2B). Nor did the vacuoles incorporate ER-Tracker, a marker for the endoplasmic reticulum (Figure 2B). Finally, the absence of labeling with MitoTracker Red (Figure 2B) indicates that the phase-lucent vacuoles were not derived from swollen mitochondria.

Although early endosomes are not sufficiently acidic to label with LysoTracker (the lumen is maintained around pH 6.2 [30]), maturation to late endosomes depends on further acidification. Bafilomycin A1 (Baf-A) is a specific inhibitor of the vacuolar-type H^+^-ATPase, which plays critical role in the maintenance of endosomal membrane potential and lumenal pH [31]. Previous studies have shown that inhibition of H^+^-ATPase with Baf-A impedes the formation of vesicular intermediates between early and late endosomes [32]. Moreover, Baf-A strongly inhibits homotypic endosome fusion *in vitro *[33], blocks endosomal vacuolization induced by *H. pylori *[34], and disperses giant endosomes generated in cells expressing constitutively active Rab 5 [35]. Therefore, we asked whether or not the formation of vacuoles in cells treated with MIPP requires the activity of the H^+^-ATPase. As shown in Figure 2D, short-term (1 h) incubation of U251 cells with Baf-A by itself had no morphological effect on the cells, but the inhibitor completely abrogated the ability of the cells to generate vacuoles when they were exposed to MIPP. The effect of Baf-A appeared to be specifically related to disruption of the endosomal membrane potential rather than general alkalinization of the endosomal compartment, since incubating cells with 1-5 mM ammonium chloride did not replicate the effects of Baf-A (data not shown).

**Figure 3:**
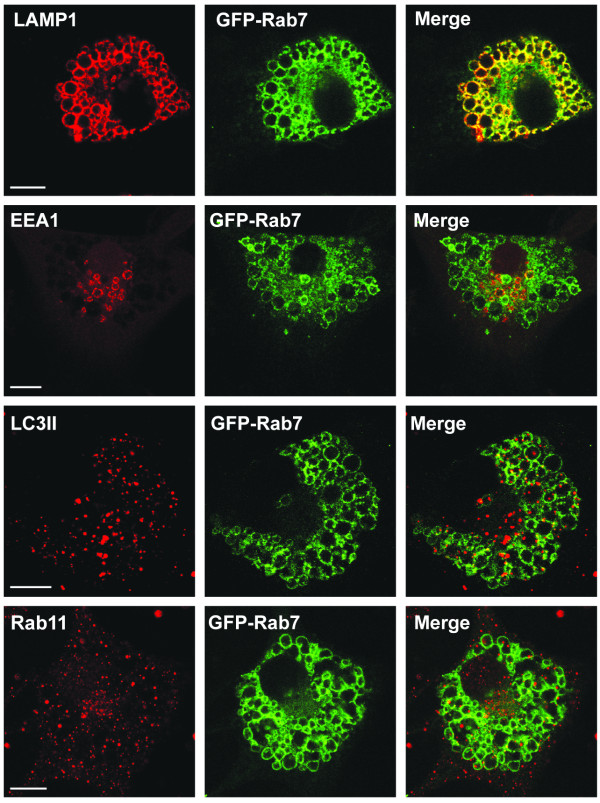
**Vacuoles induced by MIPP acquire characteristics of late endosomes, but remain distinct from autophagosomes**. U251 cells expressing GFP-Rab7 were treated with 10 μM MIPP and then processed for immunofluorescence microscopy. Confocal imaging was used to localize endogenous LAMP1, EEA1, LC3II and Rab11 (red) relative to the GFP-Rab7 (green). The scale bars are 10 microns.

Finally, we examined the distribution of markers for early and late endosomes and autophagosomes in cells treated with MIPP. Confocal microscopy demonstrated that by 24 h essentially all of the vacuoles contained late endosomal markers, GFP-Rab7 and LAMP1, but showed little or no overlap with early endosomes (EEA1), recycling endosomes (Rab11), or autophagosomes (LC3II) (Figure 3). Interestingly, the presence of the late endosome marker, Rab7, was detected in membranes of the vacuoles as soon as 30 min after the addition of MIPP, whereas the early endosome marker, Rab5, was generally absent from the majority of the vacuoles (Additional file 3, Fig. S1). Taken together with the previous studies, these observations support the idea that cellular vacuolization induced by MIPP involves fusion of nascent macropinosomes to form large vacuoles that rapidly mature to acquire some characteristics of late endosomes. However, these structures do not merge with lysosomes or autophagosomes.

### MIPP Affects the Activities of Endosomal Rab GTPases, but not Rac1 or Arf6

**Figure 4:**
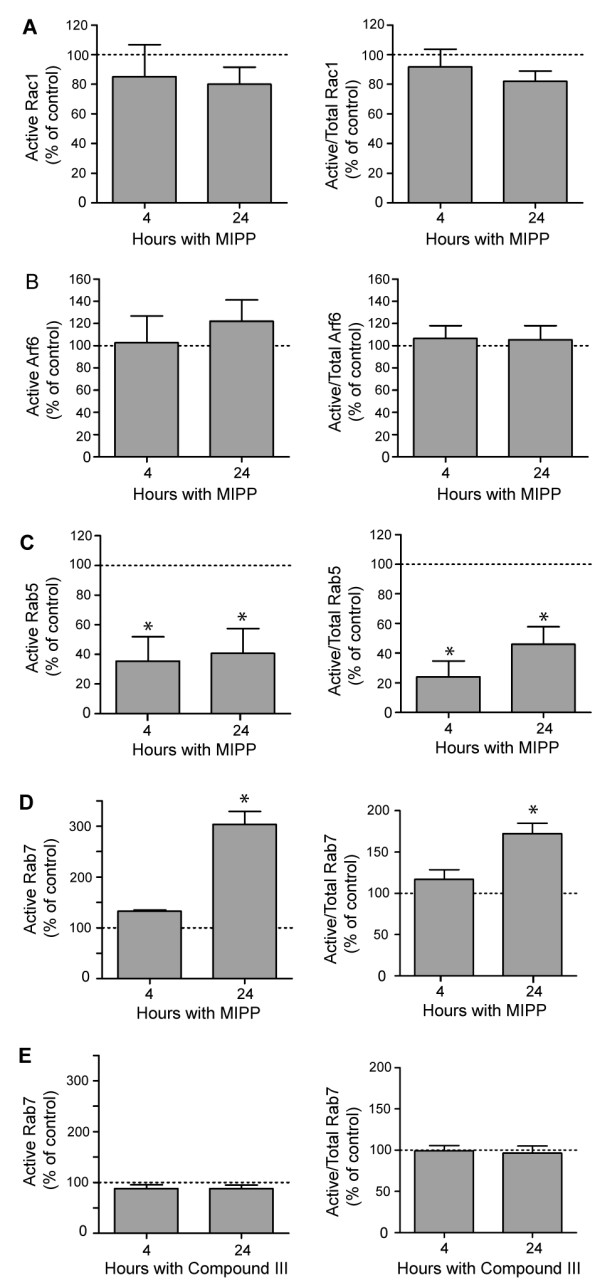
**MIPP affects the activation states of Rab5 and Rab7, but not Rac1 or Arf6**. In separate experiments U251 cells were treated with 10 μM MIPP for the indicated periods of time and then harvested for pull down assays to measure the relative amounts of active Rac1 (A), Arf6 (B), Rab5 (C) or Rab7 (D), as described in Materials and Methods. In each case the values for active GTPase (left graph) or active/total GTPase (right graph) were expressed as the percent of the corresponding values for parallel control cultures that received an equivalent volume of vehicle (DMSO), indicated by the dashed line in each graph. As an additional control, the studies of Rab7 were conducted with cells treated with the inactive compound III instead of MIPP (E). Results are the means (± SD) compiled from three separate experiments. Values marked with an asterisk were significant at p ≤ 0.05 compared with the controls.

To begin to explore the molecular mechanism(s) through which MIPP causes endosomal vacuolization, we considered possible parallels with the mechanism of methuosis triggered by over-expression of activated H-Ras. In the latter case, we found that development of the vacuolar phenotype requires activation of the Rac1 GTPase, with a concomitant reduction in the activation state of another GTPase, Arf6 [17]. However, when fusion proteins that bind specifically to the activated forms of Rac1 or Arf6 were used in pull-down assays to measure the activation states of these GTPases, we found that treatment of cells with MIPP had no significant effects on the amounts of active Rac1 (Figure 4A) or Arf6 (Figure 4B) at either 4 h or 24 h after addition of the compound. Consistent with this observation, we determined that when U251 cells were incubated with MIPP in the presence of EHT 1864, a highly specific Rac inhibitor [36], there was no detectable effect on vacuole formation (Additional file 4, Fig. S2). These studies indicate that the mechanism of vacuolization induced by MIPP is different from that induced by Ras, in that it does not depend on activation of the Rac1 signaling pathway.

In light of the striking effects of MIPP on the clathrin-independent endosomal compartment, we next focused on the Rab5 and Rab7 GTPases, which are known to function in early and late endosomal trafficking steps, respectively [37]. The active GTP-bound forms of these proteins were measured in pull-down assays using GST-fusion constructs containing the Rab binding domains of rabaptin-5, for Rab5 [38], or RILP, for Rab7 [39]. As shown in Figure 4C, MIPP caused a significant decline in the amount of active Rab5 at both 4 h and 24 h after treatment. In contrast, MIPP had the opposite effect on Rab7, with the amount of active Rab7 more than doubling by 24 h (Figure 4D). The change in active Rab7 at 24 h was moderated somewhat (but still significant) when the results were normalized to the total Rab7 pool. This is due to the fact that the total amount of Rab7 in the MIPP treated cells increased substantially by 24 h. It is important to note that the changes in the Rab activity were observed only in cells treated with the vacuole-inducing compound, MIPP. For example, in cells treated with the related but inactive compound III (see Figure 1), there was no change in the activation state of Rab7 (Figure 4E). In summary, these results suggest that the vacuolization of macropinosome-derived endocytic compartments in MIPP-treated cells is associated with opposite changes in the pools of active Rab5 and Rab7.

To determine if the initial MIPP-induced decrease in active Rab5 is the primary defect responsible for the chain of events that leads to methuosis, we over-expressed constitutively active GFP-Rab5(Q79L) in cells treated with MIPP to see if it might compensate for the loss of endogenous Rab5-GTP. The results indicate that Rab5(Q79L) was unable to prevent MIPP-induced vacuolization or restore cell viability (Additional file 5, Fig. S3). A caveat is that Rab5(Q79L) itself has a tendency to promote the formation of giant endosomes by triggering homotypic fusion of early endosomes, as noted in previous reports [35,40]. However, these structures never become as large or as numerous as the vacuoles induced by MIPP. In any case, the results show that simply augmenting the intracellular pool of active Rab5 is not sufficient to block the effects of MIPP. Similarly, overexpression of dominant-negative GFP-Rab7(N125I) to counteract the increase in endogenous Rab7-GTP also failed to prevent methuosis induced by MIPP (Additional file 5, Fig. S3). This supports the idea that the accumulation of active Rab7 is a consequence rather than a direct cause of the endosomal trafficking defects triggered by MIPP.

### MIPP Induces Non-Apoptotic Cell Death with the Characteristics of Methuosis

**Figure 5:**
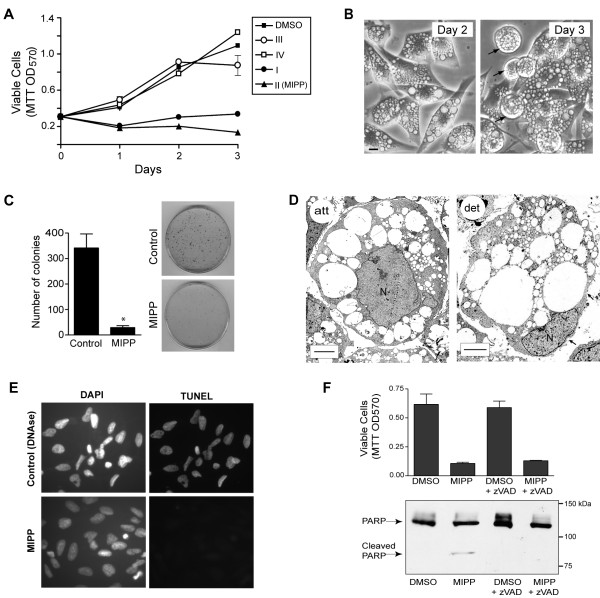
**MIPP-induced vacuolation leads to non-apoptotic cell death in glioblastoma cells**. A) MTT assay of U251 cells treated over time with the indicated compounds (refer to Fig. 1 for structures) at a concentration of 10 μM. Each point represents the mean (± SD) of results from quadruplicate wells. The decreases in viability of the cells treated with MIPP (▲) or compound I (●) were significant at p < 0.001 on days 2 and 3. B) Phase-contrast images of U251 cells treated for 2 or 3 days with 10 μM MIPP. The arrows point to vacuolated cells that have rounded and detached from the surface of the dish. The scale bar is 10 microns. C) U251 cells were treated with 10 μM MIPP or an equivalent volume of DMSO (control) for 2 days and colony forming assays were performed as described in Materials and Methods. The results shown are the mean (± SD) of triplicate dishes, with representative dishes shown at the right. The decrease in the number of colonies for the MIPP-treated cells (*) was significant at p < 0.001. D) U251 cells were examined by electron microscopy after two days of treatment with 10 μM MIPP. The left panel (att) is a representative image of an attached cell and the right panel (det) is representative of a cell that had detached from the dish. The arrows point to regions of plasma membrane discontinuity indicative of cell rupture. Nuclei (N) do not show changes in chromatin distribution typical of apoptosis. The scale bars are 10 microns. E) U251 cells treated with 10 μM MIPP for 2 days are negative for TUNEL staining. F) Inhibition of caspase activity does not prevent MIPP-induced cell death. U251 cells were seeded at 350,000 cells per 60 mm dish. The next day cells were treated with 10 μM MIPP or an equivalent volume of DMSO, in the presence or absence of 50 μM z-VAD-fmk. After two days the attached and detached cells were pooled and harvested for immunoblot analysis of PARP. MTT assays were performed on cells treated in the same manner, except that they were seeded in a 96-well plate. Values are means (± SD) of quadruplicate samples.

**Figure 6:**
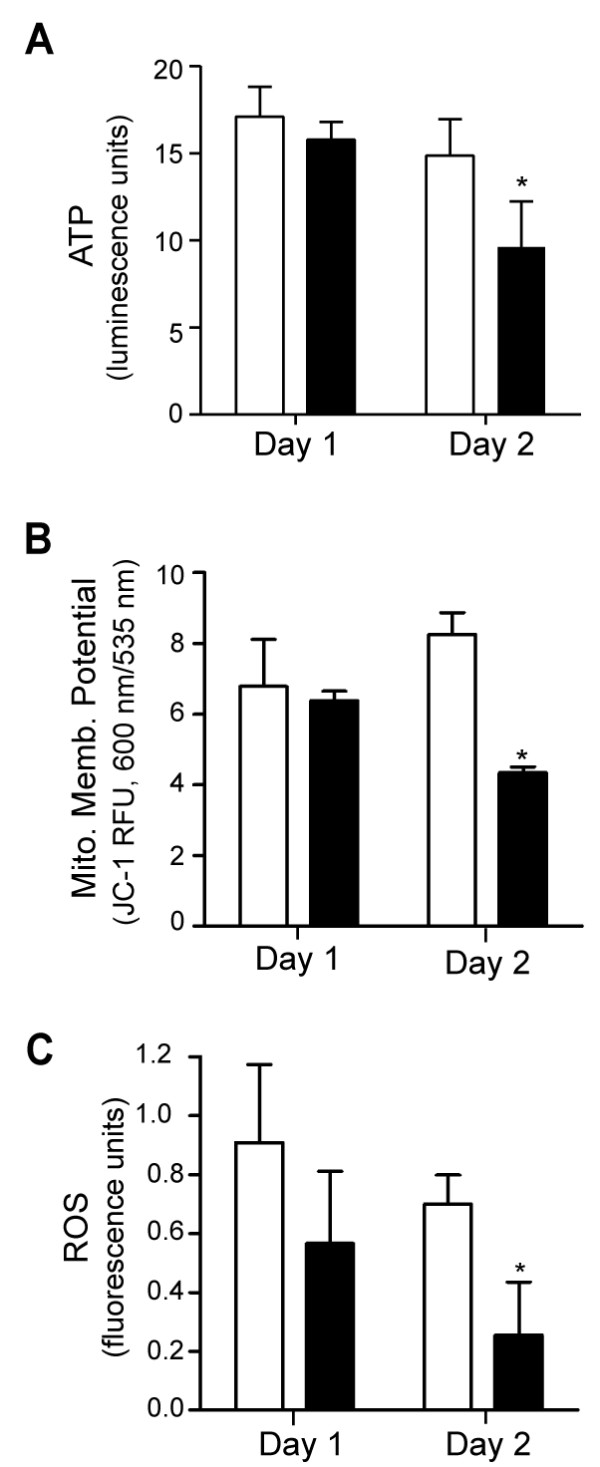
**Deficiency of mitochondrial energy metabolism accompanies cell death in cells treated with MIPP**. For all graphs the solid bars are values for cells treated with 10 μM MIPP and the open bars are controls treated with an equivalent volume of DMSO. A) ATP levels were measured as described in the Methods. The decrease in ATP in MIPP-treated cells on day-2 (*) was significant at p < 0.02 compared to the cells treated with DMSO. B) Mitochondrial membrane potential was evaluated using the JC-1 assay. The significant decline (p < 0.0001) in the ratio of fluorescence emission at 600 nm vs. 535 nm on day-2 signifies a disruption of mitochondrial membrane potential. C) There is no increase in intracellular ROS associated with MIPP-induced cell death, based on measuring the fluorescence of the ROS indicator, H_2_DCFDA. The decline in ROS in the MIPP-treated cells on day-2 was significant at p < 0.005.

We next conducted a series of studies to determine how closely the sequelae of MIPP treatment match the cell death phenotype associated with methuosis. As reported previously [16], the initial accumulation of vacuoles in glioblastoma cells undergoing Ras-induced methuosis is followed by a decline in cellular ATP levels, cell rounding, and detachment of cells from the substratum. Cell death ensues as the vacuoles expand to fill most of the cytoplasmic space and cell membrane integrity is disrupted. Characteristically, these alterations are *not *accompanied by morphological changes typical of apoptosis, such as nuclear chromatin condensation, nuclear fragmentation or cell shrinkage. Although caspase activation can be detected by examining PARP cleavage, cell death by methuosis cannot be prevented by treatment with caspase inhibitors. The studies described in Figures 5 and 6 indicate that the form of cell death induced by MIPP in U251 glioblastoma cells shares all of these characteristics.

As shown in Figure 5A, both of the vacuole-inducing compounds, MIPP and compound I, caused a marked decrease in cell growth and relative cell viability, measured by MTT assay, during the first two days of treatment. In contrast, the structurally related compounds III and IV that did not cause cellular vacuolization (Figure 1) had little effect on cell growth/viability during the same period (Figure 5A). By the second day after addition of MIPP, the cells exhibited a significant decrease in the level of ATP and a decline in mitochondrial membrane potential (Figure 6A &6B), indicative of metabolic compromise.

However, there was no general increase in reactive oxygen species in the MIPP-treated cells (Figure 6C). On the contrary, the level of ROS in the MIPP-treated cells was significantly lower than in the controls.

We noted that the number of rounded and detached cells increased dramatically in the MIPP-treated cultures between days 2 and 3 (Figure 5B). Cells that had been treated with 10 μM MIPP for 2 days were essentially non-viable when evaluated in colony-forming assays (Figure 5C). Further evaluation of the MIPP-treated cells by electron microscopy revealed that both the attached and detached cells contained numerous large, mostly empty, vacuoles bounded by a single membrane (Figure 5D). These structures were indistinguishable morphologically from the vacuoles induced by activated Ras in previous studies [16]. In the detached cells, the vacuoles had typically expanded to the point where they displaced much of the cytoplasmic volume and, in many cases, the plasma membrane was disrupted (Figure 5D, arrows). Of particular note, even in highly vacuolated cells that appeared on the verge of lysis, the nuclear chromatin remained diffuse and the nuclear membrane appeared intact (Figure 5D). Consistent with this observation, staining the MIPP-treated cells by TUNEL did not reveal evidence of nucleosomal DNA fragmentation (Figure 5E). These observations were suggestive of a non-apoptotic death mechanism. To confirm this, we asked whether the broad spectrum caspase inhibitor, zVAD-fmk, could prevent cell death in U251 glioblastoma cells treated with MIPP (Figure 5F). Similar to our previous observations with Ras-induced methuosis, the dying cells treated with MIPP showed some evidence of caspase activation (*i.e*., cleavage of full-length PARP to an 82 kDa fragment). However, caspase activation was not necessary for cell death. That is, even though zVAD-fmk was able to block PARP cleavage by caspase, it did not prevent the loss of viability in the MIPP-treated cells (Figure 5F). When combined with the lack of a morphological signature for apoptosis, these findings support our hypothesis that MIPP-induced cell death is independent of caspase activation and is due mainly to metabolic crisis and physical disruption of the highly vacuolated cells.

Since brief treatment with 100 nM Baf-A was able to impede the initial formation of vacuoles in cells treated with MIPP (Figure 2D), we considered the possibility that continued incubation of U251 cells in medium with Baf-A might counteract the cytotoxic effects of MIPP. However, these studies were hindered by the confounding effects of Baf-A itself on endosomal trafficking, which can result in vacuolization of late endosomes [41] and inhibition of cell growth and viability [42]. We found that prolonged incubation of U251 cells with 50 nM Baf-A alone caused accumulation of vacuoles by 24 h and eventually led to cell death (Additional file 6, Fig. S4).

### MIPP Produces Similar Effects in a Broad Spectrum of Cancer Cells, Including Drug-Resistant Glioblastoma

To assess the possibility that the observed response to MIPP might be a unique feature of the U251 glioblastoma cell line, we examined the effects of the compound in several other cell lines. These included an additional glioma cell line (LN229), osteosarcoma cells (U2OS), and breast (MCF7), colon (SW480) and pancreatic (PANC-1) carcinoma cells. Similar to the results with U251 cells, 10 μM MIPP induced dramatic cytoplasmic vacuolization in all of the cell lines (Additional file 7, Fig. S5). Although there were some differences in sensitivity, relative cell viability determined by MTT assays was generally reduced by 50-90% in all of the cancer cell lines treated with MIPP. Colony forming assays confirmed that exposure to MIPP for 2 days significantly reduced long-term cell survival in all cases (Additional file 7, Fig. S5). In the same study we also examined the effects of MIPP on normal human skin fibroblasts and an established mammary epithelial cell line (MCF-10A). Although these cell lines also underwent extensive cytoplasmic vacuolization, the reductions in cell viability (30% for MCF-10A and 40% for fibroblasts) were more moderate than what we observed for the cancer cell lines.

**Figure 7:**
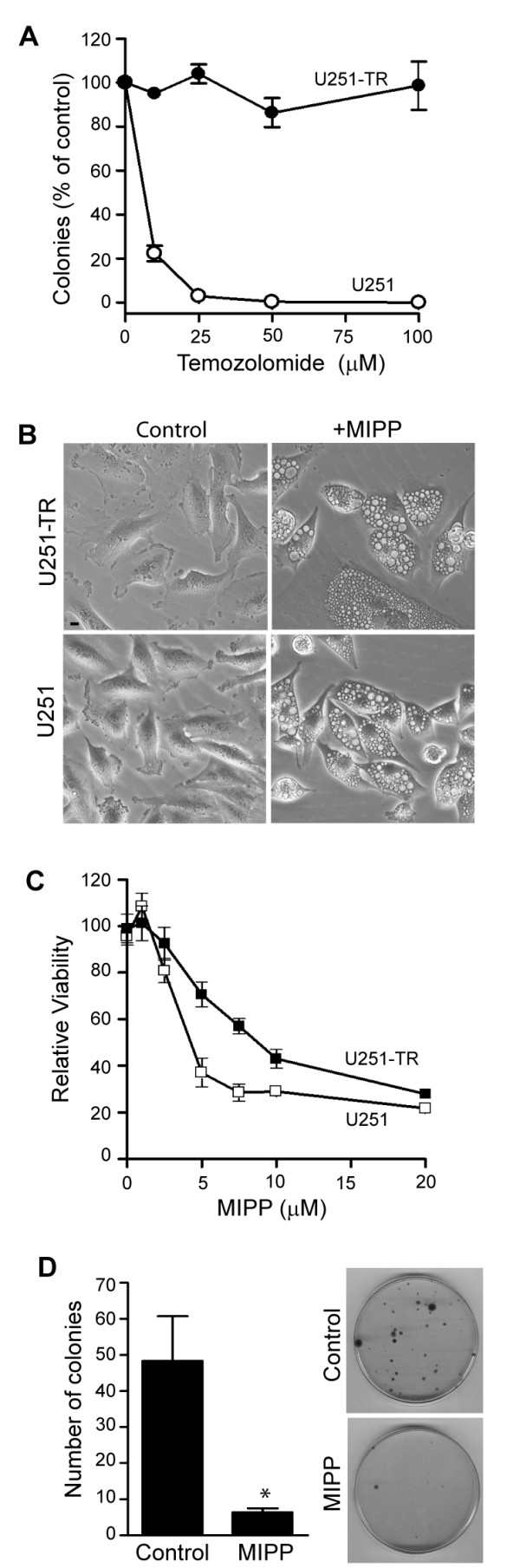
**MIPP has similar effects on cell morphology and viability in temozolomide-resistant (U251-TR) and parental (U251) glioblastoma cells**. A) Cells were treated with the indicated concentrations of TMZ for 48 h. Colonies were counted as described in Materials and Methods and the results were expressed as the percentage of the mean value for parallel control plates for the same cell lines without drug. Each point is the mean from three plates (± SD). B) Phase-contrast images were obtained after treatment of cells for 48 h with 10 μM MIPP or an equivalent volume of DMSO (control). C) MTT assays were performed after treatment for 48 h with the indicated concentrations of MIPP or an equivalent volume of DMSO. The quadruplicate MTT values for the MIPP-treated wells were expressed as percent of the mean for the parallel DMSO-treated wells. Relative IC_50 _values were calculated from nonlinear regression analysis of sigmoidal dose response curves plotted with GraphPad Prizm 4.0 (La Jolla, CA). D) U251-TR cells were treated with 10 μM MIPP or an equivalent volume of DMSO (control) for 2 days and colony forming assays were performed. The results shown are the mean (± SD) of triplicate dishes, with representative dishes shown at the right. The decrease in the number of colonies for the MIPP-treated cells (*) was significant at p < 0.001.

Our initial interest in MIPP and related compounds was based on the premise that because they induce cell death by a non-apoptotic mechanism, involving perturbations of vesicular trafficking rather than DNA replication/repair, they might be useful against cancers that have acquired resistance to drugs that work by damaging DNA. To test this hypothesis, we derived temozolomide-resistant clones from the U251 glioblastoma cell line. The survival study depicted in Figure 7A shows an example of one such clone (U251-TR), which was highly resistant to TMZ in comparison to the parental U251 cell line. When U251-TR cells were treated with 10 μM MIPP, they underwent extensive vacuolization identical to that observed in the parental U251 cells (Figure 7B). MTT dose-response curves indicated that both the parental U251 and U251-TR cells were sensitive to MIPP over a 2-day period (Figure 7C), although the relative IC_50 _value for the resistant cells (6.0 μM) was slightly higher than for the parental cells (3.5 μM). As in the case of the parental U251 cells (Figure 5C), treatment of the U251-TR cells resulted in a significant decline in survival, assessed by colony forming assays (Figure 7D). Similar results were obtained with additional TMZ-resistant clones (not shown).

## Discussion

Glioblastomas are highly aggressive brain tumors that almost always recur after surgery. Treating these tumors is extremely challenging because the residual cells are highly invasive [43] and they typically harbor genetic mutations that decrease their sensitivity to apoptosis [44,45]. The mainstays for adjuvant therapy of glioblastoma are radiation and TMZ [46]. TMZ has been reported to cause senescence and apoptosis in glioblastoma [47], but sensitivity to TMZ-induced apoptosis is blunted in cells with tumor suppressor mutations (*e.g*., p53) [48]. There is some evidence that TMZ may be able to overcome the intrinsic resistance of glioblastoma to apoptosis by inducing autophagic cell death [49,50]. Nevertheless, glioblastomas ultimately develop resistance to TMZ through a combination of factors, including alterations in DNA repair capacity [51,52]. The discovery of novel cell death mechanisms that do not depend on DNA damage could present new opportunities to treat these devastating tumors. Along sthese lines, Lefranc *et al*. [53] have reported that targeting the α1 subunit of the Na^+^/K^+^-ATPase, which is highly expressed in glioblastoma, can induce cytotoxic pro-autophagic effects in a pre-clinical model of glioblastoma. However, it is not yet known whether this approach can work against TMZ-resistant glioblastoma cells.

We recently described a unique form of cell death that is distinct from autophagy and other non-apoptotic forms of death. This form of death, termed methuosis, can be triggered by ectopic expression of constitutively activated Ras in glioblastoma and other cancer cell lines [16,17]. In this study we have identified a small molecule termed MIPP, which is capable of inducing the hallmark cytopathological features of methuosis by directly interfering with vesicular trafficking in the endocytic pathway. Shortly after being exposed to MIPP, glioblastoma cells exhibit a massive influx of macropinocytotic vesicles. In our time-lapse studies, the latter can be seen undergoing fusion events to form larger vacuoles that rapidly acquire late endosomal characteristics (Rab7, LAMP1). However, the vacuoles do not appear to merge with lysosomal compartments. Ultimately, displacement of much of the cytoplasmic space by the accumulated vacuoles is accompanied by a decline in metabolic activity and rupture of the cell. Consistent with a non-apoptotic mechanism, these changes cannot be prevented by caspase inhibitors, and nuclear chromatin condensation and TUNEL staining typical of apoptosis are not observed.

A key finding is that glioblastoma cells selected for resistance to TMZ are susceptible to MIPP-induced methuosis. We have recently obtained similar results comparing doxorubicin-resistant *versus *non-resistant MCF-7 breast cancer cells treated with a structural analog of MIPP (to be described in a separate report). This raises the intriguing possibility that MIPP may serve as a prototype for development of drugs that could be used to trigger death by methuosis in drug-resistant cancers. Although compounds related to MIPP could have potential as novel therapeutic agents, MIPP itself may not be an ideal choice for *in vivo *testing at the present time. One of the problems we have noted is the limited solubility of the compound in aqueous solutions. We are currently attempting to address this issue by synthesizing a directed library of related compounds to better understand the structure-activity relationships and to identify more potent derivatives with more versatile solubility properties. In this regard, the initial comparisons of MIPP with the inactive compounds included in this report, as well as with other unpublished analogs, provide some helpful insights. For example, activity seems to require the presence of both an electron-rich heteroaryl system (the indole ring) and an electron deficient heteroaryl system (the pyridine ring). Modifications to either ring structure can abolish activity, even if the chalcone-like 2-propen-1-one bridge is retained. This level of structural specificity argues against the idea that MIPP might serve as a broad-spectrum Michael inhibitor, and indicates that the effects of MIPP on endocytic vesicular trafficking and cell viability are most likely related to interactions with specific intracellular protein targets.

**Figure 8:**
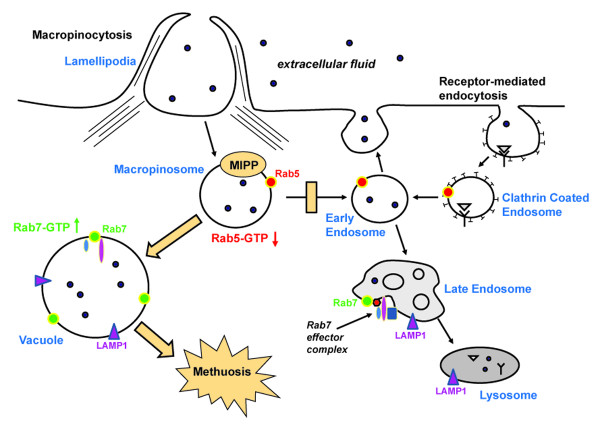
**A working model for MIPP-induced methuosis suggested by the present studies: The earliest visible effect of the compound involves stimulation of macropinosome influx**. Interaction of MIPP with unidentified components of the nascent macropinosomes results in a decline in active Rab5 and deficient delivery of macropinosomes to the early endosome sorting compartment. This precludes recycling or maturation to normal multivesicular and late endosomes. The abnormal macropinosomes undergo homotypic fusion and rapidly acquire some characteristics of late endosomes (e.g., LAMP1 and Rab7). However, they apparently lack key molecular components required for fusion with lysosomes. Consequently, they accumulate as vacuoles and eventually fill the cell, impairing metabolic function (decrease in ATP and mitochondrial membrane potential) and disrupting membrane integrity.

Clues concerning the steps involved in MIPP-induced methuosis come from a consideration of the pathways for macropinocytosis in mammalian cells. Macropinosomes are normally internalized through an actin-dependent process that requires sequential recruitment of phosphatidylinositol 4,5-bisphosphate and phosphatidylinositol 3,4,5-trisphosphate [54,55]. Once internalized, macropinosomes rapidly acquire Rab5, EEA1 and sorting nexins, which promote fusion with early endosomes [56,57]. The latter retain the capacity to recycle to the cell surface [55], but after several additional minutes they lose Rab5 and acquire Rab7 and LAMP1, taking on the characteristics of late endosomes [57,58]. Ultimately, the fluid-filled vesicles dissipate as they merge with lysosomes [58]. Our finding that MIPP initially causes a significant decrease in the activity of Rab5, followed by an increase in the activity of Rab7 suggests a working model depicted in Figure 8. We hypothesize that MIPP targets one or more proteins in endosomal complexes required for fusion of nascent macropinosomes with early endosomes, directly or indirectly affecting the guanine nucleotide cycle of Rab5. This prevents the vesicles formed during the initial burst of macropinocytotic activity from entering the early and recycling endosomal compartments. This is consistent with the general absence of Rab5, EEA1 and Rab11 from the vacuoles.

The vesicles that bypass the normal endosomal trafficking pathway immediately recruit Rab7 and begin to undergo abnormal homotypic fusions to form progressively larger LAMP1-positive structures (Figure 8). Precedent for such an endosomal 'bypass' mechanism has recently been described for a macropinocytosis-like pathway used by BTV-1 virus to infect BHK cells. In this system, clathrin-independent vesicles carrying the virus rapidly merge with LAMP1-positive late endosomes without passing through early or recycling endosome compartments [59]. It remains unclear why the Rab7-positive vacuoles are able to fuse with each other but cannot merge with lysosomes or autophagosomes. One possibility is that in bypassing the normal endosomal trafficking route the vacuoles fail to acquire key proteins needed for heterotypic tethering and fusion with lysosomes (*e.g*., HOPS, *trans*-SNARE) [60,61]. This model could also explain the eventual increase in the amount of Rab7-GTP, as GAP-mediated GTP-hydrolysis that normally accompanies endosome-lysosome fusion would fail to occur. An important goal for the future will be the precise identification of the molecular targets of MIPP. Given the efficacy of MIPP against TMZ-resistant cells, possible candidates might be found among the list of genes recently shown to be induced in TMZ-resistant glioblastoma cells [62]. However, because of the complexity of the trafficking pathways underlying the methuosis phenotype, we believe that unbiased approaches to drug target discovery will stand the best chance of defining the relevant targets of the compound.

Based on our examination of several cancer cell lines, it seems clear that the ability of MIPP to induce methuosis extends beyond glioblastoma. While broad-spectrum activity against a variety of cancers ultimately may prove to be a positive attribute, the observation that MIPP can also trigger vacuolization and a modest reduction of cell proliferation/viability in normal proliferating cells, raises a note of caution. Eventual testing of MIPP-related compounds *in vivo *may need to incorporate approaches for selective delivery of such compounds to tumors. In this regard, recent advances in tumor targeted drug delivery strategies [63-65] should provide a number of options. Given the potential value of a therapeutic agent that can induce non-apoptotic death in drug-resistant cancer cells, we believe that further development of MIPP-related compounds, identification of their specific molecular targets, and investigation of their therapeutic properties are worthwhile goals.

## Methods

### Test Compounds and Reagents

Compounds I, II (MIPP), and IV were purchased from Chembridge Corporation (San Diego, CA). Compound III was purchased from TimTec, LLC (Newark, DE). Each of these compounds was stored at-20°C as a 5 mg/ml stock solution in DMSO, and then diluted to the indicated final concentration in cell culture medium. Filipin, bafilomycin A1 and temozolomide (TMZ) were obtained from Sigma-Aldrich (St. Louis, MO). Filipin was stored at -20°C as a 50 mg/ml stock in DMSO and bafilomycin A1 was stored at -20°C as a 10 μM stock in DMSO. EHT 1864 was generously provided by Exonhit Therapeutics, Paris, France. z-VAD-fmk was purchased from Bachem (Torrance, CA).

### Cell Culture

U251 human glioblastoma cells were purchased from the DCT Tumor Repository (National Cancer Institute, Frederick, MD). All other cell lines were obtained from the American Type Culture Collection (Rockville, MD). Cell lines were passaged for fewer than six months prior to use. Normal human skin fibroblasts were derived from a skin biopsy as described previously [66]. Unless stated otherwise, cell lines were maintained in Dulbecco's modified Eagle medium (DMEM) with 10% (v/v) fetal bovine serum (FBS) (JR Scientific, Woodland, CA) at 37°C with 5% CO_2_/95% air. MCF-10A cells were maintained in DMEM + Ham's F12 (1:1) containing 5.0% horse serum, 20 ng/ml EGF, 0.5 μg/ml hydrocortisone, 100 ng/ml cholera toxin, and 10 μg/ml insulin, as described [67]. Colonies of TMZ-resistant U251 cells were selected by maintaining the parental U251 cells in medium containing 100 μM TMZ for 24 days, with replenishment of the medium and drug every three days. Colonies were then picked and plated into a 12-well dish and exposed to escalating concentrations of TMZ. The clone used in the present study (U251-TR) is maintained routinely in medium with 300 μM TMZ. Phase-contrast images of live cells were obtained using an Olympus IX70 inverted microscope equipped with a digital camera and SPOT imaging software (Diagnostic Instruments, Inc., Sterling Heights, MI).

### Live Cell Imaging with Fluorescent Tracers

Lucifer yellow (LY) was purchased from Invitrogen/Molecular Probes (Carlsbad, CA). Labeling of endocytic compartments with this fluid-phase tracer was performed as previously described [16]. Labeling of intracellular acidic compartments with LysoTracker Red DND-99 (Invitrogen) and staining for cathepsin B activity with Magic Red RR (ImmunoChemistry Technologies, Bloomington, MN) were performed as described previously [16]. ER-Tracker Blue-White DPX (Invitrogen) was used to label the endoplasmic reticulum following the directions supplied by the manufacturer. Cells were incubated with 200 nM MitoTracker Red CMXRos (Invitrogen) in Hank's balanced salt solution for 20 min to label the mitochondria. Phase-contrast and fluorescent images of the living cells were acquired on an Olympus IX70 inverted microscope equipped with a digital camera and SPOT imaging software or on a Nikon Eclipse TE2000U fluorescence microscope with a digital camera and NIS-Elements AR software (Nikon Instruments, Inc., Melville, NY).

### Time-Lapse Microscopy

200,000 U251 cells were plated in a 35 mm glass-bottom microwell culture dish (MatTek, Ashland, MA). The day after plating, the cells were treated with 10 μM MIPP in phenol red-free DMEM supplemented with 10% FBS. The dish was immediately placed in a humidified Live Cell chamber (Pathology Devices, Westminster, MD) equilibrated with 5% CO_2 _at 37°C. The chamber was placed on the stage of an Olympus IX80 inverted microscope, equipped with a digital camera and Slidebook software (Intelligent Imaging Innovations, Inc., Denver, CO). The software was set to automatically acquire phase-contrast images every 30 sec for the indicated period of time.

### Treatment of Cells with Filipin, Bafilomycin A1 or EHT 1864

To inhibit clathrin-independent endocytosis, U251 cells were washed twice with serum-free DMEM, then pretreated for 30 min with DMEM + 0.5% BSA in the presence or absence of 12 μg/ml filipin [29]. Following the pretreatment, MIPP was added to the dishes at a final concentration of 10 μM and phase-contrast images were acquired 100 min later.

To inhibit the vacuolar-type H^+ ^ATPase, U251 cells were pretreated for 1 h with 50 or 100 nM bafilomycin A1 (Baf-A) or an equivalent volume of DMSO, as indicated in the figure legends. At the end of the hour, 10 μM MIPP, or an equivalent volume of DMSO, was added without a medium change.

Phase-contrast images of the cells were acquired 1 h after the addition of MIPP. To determine the effect of MIPP and Baf-A on cell growth, 35 mm dishes were seeded with 100,000 cells one day prior to exposure to the drugs. At the indicated times after treatment, triplicate dishes of cells were harvested by trypsinization and counted with a Coulter Z1 particle counter (Beckman Coulter, Brea, CA).

To determine if inhibition of the Rac1 GTPase would block the accumulation of vacuoles, U251 cells were treated for 24 h with 10 μM MIPP in the presence or absence of 25 μM EHT 1864 (Rac inhibitor). The percentage of vacuolated cells in the population was determined by scoring at least 100 cells in multiple phase-contrast photomicrographs for each condition. Cells containing three or more phase-lucent vacuoles with diameters ≥ 0.5 μm or >10 smaller vacuoles (0.1-0.5 μm) were scored as positive. For comparison, the effect of EHT 1864 on Ras-induced vacuolization was also determined. U251 cells were nucleofected with pCMV5-Myc-H-Ras(G12V), as described previously [68], then immediately plated into medium with or without 25 μM EHT 1864. After 24 h, phase-contrast images were taken and the number of vacuolated cells was scored as described above.

### Pull-Down Assays to Measure the Activation States of GTPases

Assays for activated Rac1 and Arf6 were performed as described previously [17] using EZ-Detect Rac1 or Arf6 activation kits (Thermo Scientific Pierce, Rockford, IL). These assays employ either a GST-fusion protein containing the p21-binding domain of p21-activated protein kinase 1 (PAK1) to selectively bind active Rac1 in whole cell lysates, or GST-GGA3 (Golgi-associated gamma adaptin ear-containing Arf binding protein 3) to pull down active Arf6. The active Rac1 or Arf6 collected on the glutathione beads were subjected to western blot analysis and the chemiluminescence signals were quantified using Alpha Innotech FluorChem HD2 imaging system. The values for active protein in each sample were normalized to α-tubulin. Results were expressed as either the total active Rac1 or Arf6, or the ratio of the active GTPase to the total Rac1 or Arf6 measured in aliquots of the cell lysate.

Prokaryotic expression vectors encoding GST-RILP or GST-Rabaptin-5 (C-terminus) were kindly provided by Cecilia Bucci (University of Salento, Italy) and José A. Esteban (Universidad Autónoma de Madrid, Spain), respectively. GST-fusion proteins were produced in *E. coli *BL21 (DE3) pLysS (Promega, Madison, WI, USA) and the fusion proteins were bound to glutathione-sepharose 4B beads (GE Healthcare Biosciences, Pittsburgh, PA, USA). Pull-down assays for active Rab5 using the GST-Rabaptin-5 beads were performed essentially as described by Brown *et al*. [38]. Assays for active Rab7 were performed as described by Romero Rosales *et al*. [69]. For each determination cell lysates were prepared from ten pooled 10 cm cultures. Monoclonal antibodies against Rab5 or Rab7 (Cell Signaling Technologies, Danvers, MA) were used to probe western blots of the proteins collected on the beads. Results were quantified and normalized essentially as described above for Rac1 and Arf6.

### Effects of Dominant-Negative or Constitutively Active Rab Constructs on Methuosis

pEGFP-C1 was purchased from Clontech (Mountain View, CA), pEGFP-Rab5(Q79L) was provided by Guangpu Li, University of Oklahoma College of Medicine, and pEGFP-Rab7(N125I) was provided by Cecilia Bucci, University of Salento. U251 cells were nucleofected with pEGFP-C1, pEGFP-Rab5(Q79L) or pEGFP-Rab7(N125I) as previously described [68]. The cells were plated either in 35 mm dishes for live cell imaging or 96-well plates for MTT assay. The day after nucleofection, the cells were treated with 10 μM MIPP or an equivalent volume of DMSO and cells were monitored by MTT assays or phase contrast and fluorescence microscopy.

### Confocal Fluorescence Microscopy

U251 cells were nucleofected with pEGFP-Rab7 or pEGFP-Rab5, then plated onto coverslips in 60 mm dishes. The day after nucleofection, the cells were treated with 10 μM MIPP. For colocalization experiments, cells that had been treated with MIPP for 24 h were prepared for immunofluorescence microscopy as described previously [70]. Antibodies to detect endogenous LAMP1 (Developmental Studies Hybridoma Bank, University of Iowa, Iowa City, IA), EEA1 (Abcam, Cambridge, MA), LC-3 (Abgent, Inc., San Diego, CA), and Rab11 (Invitrogen) were obtained from the indicated sources. In a previous study we found that the LC3 antibody predominantly recognizes the autophagosomal form of LC3, LC3II [16]. All primary antibodies were detected by incubation with goat anti-mouse IgG conjugated to Alexa Fluor 568 (Invitrogen). Cells were examined by confocal microscopy using a Leica TCS SP5 system with 488- and 561-nm laser excitation. Images were acquired with the LASAF software on the system.

### Electron Microscopy

U251 cells were exposed to 10 μM MIPP for 48 h, then prepared for electron microscopy as described previously [70]. The cells were examined under a Philips CM 10 transmission electron microscope.

### Western Blot Analysis

The antibody for PARP was purchased from BD Biosciences (San Jose, CA). Methods for protein determination, SDS-PAGE and western blot analysis have been described previously [71].

### Cell Viability

Cell viability was measured using a 3-(4,5-dimethylthiazol-2-yl)-2,5-diphenyl tetrazolium bromide (MTT)-based assay. A 5 mg/ml MTT (Amresco, Solon, OH) stock solution was prepared in phenol-red free RPMI 1640 (Mediatech, Inc, Herndon, VA). Cells were seeded in 96-well plates, with four replicate wells for each culture condition. On the day of the assay, 10 μl of the MTT solution was added directly to 100 μl in each well and the cells were incubated for 3-4 h at 37°C, with 5% CO_2_. At the end of the incubation, 100 μl of MTT solvent (0.1N HCl in isopropanol, containing 0.1% NP-40) was added to each well. The plates were incubated for an additional 5 min at 37°C, 5% CO_2_, then quantified for absorbance at 570 nm on a SpectraMax Plus 384 plate reader (Molecular Devices, Sunnyvale, CA).

For colony-forming assays, cells were plated in 100 mm dishes at 2,500 (U251 and U251-TR) or 1,500 (all other cell lines) cells per dish. Beginning on the day after plating, the cells were exposed to 10 μM MIPP for 2 days, with the medium and drug replenished after 1 day. The cells were fed fresh medium without drug every 2 to 3 d for a period of 10-21 days. To visualize the colonies, the dishes were washed with PBS, fixed for 10 min with ice-cold 100% methanol, and stained with 1% crystal violet (Acros Organics, Fisher Scientific, Pittsburgh, PA) in 35% methanol. After 2-3 washes with water, colonies containing at least 50 cells were counted using a dissecting microscope or a Protocol 2 colony counter (Synbiosis, Frederick MD).

Nuclear DNA fragmentation resulting from apoptotic cell death was assayed using the DeadEnd Fluorometric TUNEL System from Promega according to the manufacturer's protocol, including the use of DNAse I treatment as a positive control. U251 cells were seeded at 500,000 cells in 60 mm dishes containing a coverslip. Treatment with 10 μM MIPP or an equal volume of DMSO began one day after plating, with medium and drug being replenished each day until the TUNEL assay was performed.

To compare the levels of ATP in MIPP-treated glioblastoma cells versus controls treated with an equivalent volume of DMSO, the cells were harvested by trypsinization and assayed using the CellTiter Glo kit from Promega (Madison, WI) according to the manufacturer's instructions. The effect of MIPP treatment on the electrochemical gradient across the mitochondrial membrane was determined using the JC-1 Mitochondrial Membrane Potential Detection Kit from Biotium (Hayward, CA), following the manufacturer's recommended protocol. Reactive oxygen species (ROS) were measured using the 5-(and -6)-carboxy-2',7'-dichlorodihydrofluorescein diacetate (H_2_DCFDA) reagent from Invitrogen. U251 cells were seeded in phenol red-free DMEM + 10% FBS at 5,000 cells per well in black 96-well plates the day before treatment. Quadruplicate wells were treated with 10 μM MIPP, or an equal volume of DMSO. At the indicated times, the medium was removed and the cells were loaded with 100 μM H_2_DCFDA in HBSS for 30 min, 37°C, 5% CO_2_. After removing the loading buffer, the cells were washed twice with HBSS, then incubated for additional 30 minutes in HBSS prior to quantification of the fluorescence.

### Statistical Significance

Statistical significance of differences in colony formation or other parameters (e.g., GTPase activation) involving comparisons between control and MIPP-treated cells were evaluated by Student's two tailed t-test.

## Abbreviations

Baf-A: bafilomycin A1; DMEM: Dulbecco's modified Eagle medium; DMSO: dimethyl sulfoxide; FBS: fetal bovine serum; GAP: GTPase activating protein; GFP: green fluorescent protein; GST: glutathione S-transferase; HBSS: Hank's balanced salt solution; LAMP1: lysosomal-associated membrane protein 1; LC3: microtubule-associated protein light chain 3; MIPP: 3-(2-methyl-1H indol-3-yl)-1-(4-pyridinyl)-2-propen-1-one; MTT: 3-(4,5-dimethylthiazol-2-yl)-2,5-diphenyl tetrazolium bromide; TMZ: temozolomide.

## Competing interests

The authors declare that they have no competing interests.

## Authors' contributions

JO designed and carried out the studies described in Figure 1, Figure 2(B, C), Figure 3, Figure 5 (A, D, E, F), Figure 6, Supplementary Movies 1 & 2, and Figs. S1, S4 and S5, and drafted the pertinent methods and figure legends. AY designed and carried out the studies described in Figure 4(C, D, E), Figure 5(B, C) and Figure 7, and drafted the pertinent methods and figure legends. HB designed and carried the studies described in Figure 4(A, B) and Supplementary Figs. S2(A, C) and S3, and drafted the pertinent methods and figure legends. WM conceived the overall line of investigation, collaborated with the other authors in the design and interpretation of all of the studies, generated Figure 8 and drafted the manuscript. All authors read and approved the final manuscript.

## Supplementary Material

Additional file 1 **Movie 1**. Quick Time movie showing the evolution of vacuoles derived from macropinosomes in U251 cells treated with 10 μM MIPP. Time-lapse phase-contrast images were captured at 30 sec intervals and assembled into a movie that covers the period between 13-80 min after the addition of the compound. Macropinosomes can be seen forming from regions of active membrane ruffling, and then fusing with each other to form larger vacuoles. The larger vacuoles migrate toward the perinuclear region, where they accumulate. Click here for file

Additional file 2 **Movie 2**. Quick Time movie showing U251 cells during the period following the initial burst of macropinocytotic activity, between 95-160 min after addition of 10 μM MIPP. Time-lapse phase-contrast images were captured at 30 sec intervals. Very few new macropinosomes form during this period. Fusions of existing vacuoles continues to occur but at a much slower rate than during the first 90 min after addition of MIPP. Click here for file

Additional file 3 **Figure S1**. U251 cells were transfected with expression vectors encoding GFP-Rab5 or GFP-Rab7. After 24 h, MIPP was added at a concentration of 10 μM and cells were examined by confocal fluorescence microscopy at the indicated intervals. The results show that even at the earliest time points most of the vacuoles are decorated with Rab7. In contrast, Rab5 is mostly localized in smaller punctate structures. The scale bars are 10 microns. Click here for file

Additional file 4 **Figure S2**. The Rac inhibitor, EHT 1864, does not block the induction of vacuoles by MIPP. U251 cells were incubated with MIPP for 24 h in the presence or absence of 25 μM EHT 1864 (panel A). In a separate experiment, U251 cells were incubated with or without EHT 1864 following nucleofection with a vector encoding a constitutively active H-Ras(G12V) (panel B). Phase-contrast images were taken 24 h after addition of the Rac inhibitor. The scale bars are 10 microns. C) The percentage of vacuolated cells for each condition was determined as described in Materials and Methods. Click here for file

Additional file 5 **Figure S3**. Expression of constitutively active Rab5 or dominant-negative Rab7 does not protect U251 cells from MIPP-induced methuosis. A) Cells were nucleofected with vectors encoding GFP or constitutively active GFP-Rab5(Q79L), as indicated above each panel. One day after nucleofection (time-0) parallel cultures were treated with 10 μM MIPP (○) or an equivalent volume of DMSO (●) and viable cells were measured by MTT assay. Cells in parallel dishes were subjected to fluorescence and phase contrast microcopy to evaluate the extent to which the nucleofected cells (green) were vacuolated. B) The identical experiment was conducted with cells expressing GFP or the dominant-negative GFP-Rab7(N125I). Click here for file

Additional file 6 **Figure S4**. Long-term treatment of U251 cells with Bafilomycin A_1 _(Baf-A) induces vacuolization and is cytotoxic. Therefore, Baf-A cannot protect cells from MIPP-induced methuosis. U251 cells were treated for 2 days with the indicated compounds. A) Cells were examined by phase contrast microscopy to assess vacuolization. B) Cell growth was assessed by counting attached cells in 3 parallel cultures (mean ± SD) at each time point. The symbols are: DMSO control (■), 10 μM MIPP alone (▲), 50 nM Baf-A alone (○), or a combination of 10 μM MIPP plus 50 nM Baf-A (●). Click here for file

Additional file 7 **Figure S5**. MIPP induces vacuoles and inhibits growth and viability in multiple human cell lines. Cell lines examined were: LN229, glioblastoma; SW480, colon adenocarcinoma; PANC-1, pancreatic carcinoma; U20S, osteosarcoma; MCF-7, mammary adenocarcinoma; MCF-10A, mammary epithelial cells; primary human skin fibroblasts. A) Phase-contrast images of cells were acquired after two days of treatment with 10 μM MIPP. The scale bars are 10 microns. B) MTT assays were performed on cells treated for the indicated number of days with 10 μM MIPP or an equivalent volume of DMSO. The MTT values for the MIPP-treated wells were expressed as percent of the mean for the parallel DMSO-treated wells. Error bars indicate the SD. C) Colony-forming assays for the transformed cell lines were preformed as described in Materials and Methods. Values are the mean (± SD) from triplicate cultures. All of the decreases in colony formation (*) were significant at p < 0.001. Click here for file

## References

[B1] Saeki T Tsuruo T Sato W Nishikawsa K Drug resistance in chemotherapy for breast cancer Cancer Chemother Pharmacol 2005 56 84 89 10.1007/s00280-005-0106-4 16273361

[B2] O'Driscoll L Clynes M Biomarkers and multiple drug resistance in breast cancer Curr Cancer Drug Targets 2006 6 365 384 10.2174/156800906777723958 16918307

[B3] Golstein P Kroemer G A multiplicity of cell death pathways. Symposium on apoptotic and non-apoptotic cell death pathways EMBO Rep 2007 8 829 833 10.1038/sj.embor.7401042 17721445 PMC1973949

[B4] Kroemer G Galluzzi L Vandenabeele P Abrams J Alnemri ES Baehrecke EH Blagosklonny MV El Deiry WS Golstein P Green DR Hengartner M Knight RA Kumar S Lipton SA Malorni W Nunez G Peter ME Tschopp J Yuan J Piacentini M Zhivotovsky B Melino G Classification of cell death: recommendations of the Nomenclature Committee on Cell Death 2009 Cell Death Differ 2009 16 3 11 10.1038/cdd.2008.150 18846107 PMC2744427

[B5] Bursch W Ellinger A Gerner C Frohwein U Schulte-Hermann R Programmed cell death (PCD). Apoptosis, autophagic PCD, or others? Ann N Y Acad Sci 2000 926 1 12 11193023 10.1111/j.1749-6632.2000.tb05594.x

[B6] Lockshin RA Zakeri Z Apoptosis, autophagy, and more Int J Biochem Cell Biol 2004 36 2405 2419 10.1016/j.biocel.2004.04.011 15325581

[B7] Gozuacik D Kimchi A Autophagy as a cell death and tumor suppressor mechanism Oncogene 2004 23 2891 2906 10.1038/sj.onc.1207521 15077152

[B8] Majno G Joris I Apoptosis, oncosis, and necrosis. An overview of cell death Am J Pathol 1995 146 3 15 7856735 PMC1870771

[B9] Trump BF Berezesky IK Chang SH Phelps PC The pathways of cell death: oncosis, apoptosis, and necrosis Toxicol Pathol 1997 25 82 88 10.1177/019262339702500116 9061857

[B10] Suarez Y Gonzalez L Cuadrado A Berciano M Lafarga M Munoz A Kahalalide F, a new marine-derived compound, induces oncosis in human prostate and breast cancer cells Mol Cancer Ther 2003 2 863 872 14555705

[B11] Degterev A Huang Z Boyce M Li Y Jagtap P Mizushima N Cuny GD Mitchison TJ Moskowitz MA Yuan J Chemical inhibitor of nonapoptotic cell death with therapeutic potential for ischemic brain injury Nat Chem Biol 2005 1 112 119 10.1038/nchembio711 16408008

[B12] Han W Li L Qiu S Lu Q Pan Q Gu Y Luo J Hu X Shikonin circumvents cancer drug resistance by induction of a necroptotic death Mol Cancer Ther 2007 6 1641 1649 10.1158/1535-7163.MCT-06-0511 17513612

[B13] Sperandio S de B Bredesen DE An alternative, nonapoptotic form of programmed cell death Proc Natl Acad Sci USA 2000 97 14376 14381 10.1073/pnas.97.26.14376 11121041 PMC18926

[B14] Wang Y Li X Wang L Ding P Zhang Y Han W Ma D An alternative form of paraptosis-like cell death, triggered by TAJ/TROY and enhanced by PDCD5 overexpression J Cell Sci 2004 117 1525 1532 10.1242/jcs.00994 15020679

[B15] Chi S Kitanaka C Noguchi K Mochizuki T Nagashima Y Shirouzu M Fujita H Yoshida M Chen W Asai A Himeno M Yokoyama S Kuchino Y Oncogenic Ras triggers cell suicide through the activation of a caspase-independent cell death program in human cancer cells Oncogene 1999 18 2281 2290 10.1038/sj.onc.1202538 10327074

[B16] Overmeyer JH Kaul A Johnson EE Maltese WA Active ras triggers death in glioblastoma cells through hyperstimulation of macropinocytosis Mol Cancer Res 2008 6 965 977 10.1158/1541-7786.MCR-07-2036 18567800 PMC2994605

[B17] Bhanot H Young AM Overmeyer JH Maltese WA Induction of non-apoptotic cell death by activated Ras requires inverse regulation of Rac1 and Arf6 Mol Cancer Res 2010 8 1358 1374 10.1158/1541-7786.MCR-10-0090 20713492 PMC2994602

[B18] Li C Macdonald JI Hryciw T Meakin SO Nerve growth factor activation of the TrkA receptor induces cell death, by macropinocytosis, in medulloblastoma Daoy cells J Neurochem 2010 112 882 899 10.1111/j.1471-4159.2009.06507.x 19943845

[B19] Nara A Aki T Funakoshi T Uemura K Methamphetamine induces macropinocytosis in differentiated SH-SY5Y human neuroblastoma cells Brain Res 2010 1352 1 10 20654590 10.1016/j.brainres.2010.07.043

[B20] Reyes-Reyes EM Teng Y Bates PJ A new paradigm for aptamer therapeutic AS1411 action: uptake by macropinocytosis and its stimulation by a nucleolin-dependent mechanism Cancer Res 2010 70 8617 8629 10.1158/0008-5472.CAN-10-0920 20861190 PMC2970734

[B21] Cerny J Feng Y Yu A Miyake K Borgonovo B Klumperman J Meldolesi J McNeil PL Kirchhausen T The small chemical vacuolin-1 inhibits Ca(2+)-dependent lysosomal exocytosis but not cell resealing EMBO Rep 2004 5 883 888 10.1038/sj.embor.7400243 15332114 PMC1299144

[B22] Go ML Wu X Liu XL Chalcones: an update on cytotoxic and chemoprotective properties Curr Med Chem 2005 12 481 499 15720256 10.2174/0929867053363153

[B23] Kamal A Ramakrishna G Raju P Viswanath A Ramaiah MJ Balakishan G Pal-Bhadra M Synthesis and anti-cancer activity of chalcone linked imidazolones Bioorg Med Chem Lett 2010 20 4865 4869 10.1016/j.bmcl.2010.06.097 20637611

[B24] Kumar D Kumar NM Akamatsu K Kusaka E Harada H Ito T Synthesis and biological evaluation of indolyl chalcones as antitumor agents Bioorg Med Chem Lett 2010 20 3916 3919 10.1016/j.bmcl.2010.05.016 20627724

[B25] Dinkova-Kostova AT Massiah MA Bozak RE Hicks RJ Talalay P Potency of Michael reaction acceptors as inducers of enzymes that protect against carcinogenesis depends on their reactivity with sulfhydryl groups Proc Natl Acad Sci USA 2001 98 3404 3409 10.1073/pnas.051632198 11248091 PMC30666

[B26] Yamakoshi H Ohori H Kudo C Sato A Kanoh N Ishioka C Shibata H Iwabuchi Y Structure-activity relationship of C5-curcuminoids and synthesis of their molecular probes thereof Bioorg Med Chem 2010 18 1083 1092 10.1016/j.bmc.2009.12.045 20060305

[B27] Swanson JA Watts C Macropinocytosis Trends Cell Biol 1995 5 424 428 10.1016/S0962-8924(00)89101-1 14732047

[B28] Grimmer S van Deurs B Sandvig K Membrane ruffling and macropinocytosis in A431 cells require cholesterol J Cell Sci 2002 115 2953 2962 12082155 10.1242/jcs.115.14.2953

[B29] Naslavsky N Weigert R Donaldson JG Characterization of a nonclathrin endocytic pathway: membrane cargo and lipid requirements Mol Biol Cell 2004 15 3542 3552 10.1091/mbc.E04-02-0151 15146059 PMC491817

[B30] Grabe M Oster G Regulation of organelle acidity J Gen Physiol 2001 117 329 344 10.1085/jgp.117.4.329 11279253 PMC2217256

[B31] Bowman EJ Siebers A Altendorf K Bafilomycins: a class of inhibitors of membrane ATPases from microorganisms, animal cells, and plant cells Proc Natl Acad Sci USA 1988 85 7972 7976 10.1073/pnas.85.21.7972 2973058 PMC282335

[B32] Clague MJ Urbe S Aniento F Gruenberg J Vacuolar ATPase activity is required for endosomal carrier vesicle formation J Biol Chem 1994 269 21 24 8276796

[B33] Hammond TG Goda FO Navar GL Campbell WC Majewski RR Galvan DL Pontillon F Kaysen JH Goodwin TJ Paddock SW Verroust PJ Membrane potential mediates H(+)-ATPase dependence of "degradative pathway" endosomal fusion J Membr Biol 1998 162 157 167 10.1007/s002329900353 9538509

[B34] Papini E Bugnoli M de Bernard M Figura N Rappuoli R Montecucco C Bafilomycin A1 inhibits Helicobacter pylori-induced vacuolization of HeLa cells Mol Microbiol 1993 7 323 327 10.1111/j.1365-2958.1993.tb01123.x 8446034

[B35] Duclos S Corsini R Desjardins M Remodeling of endosomes during lysosome biogenesis involves 'kiss and run' fusion events regulated by rab5 J Cell Sci 2003 116 907 918 10.1242/jcs.00259 12571288

[B36] Shutes A Onesto C Picard V Leblond B Schweighoffer F Der CJ Specificity and mechanism of action of EHT 1864, a novel small molecule inhibitor of Rac family small GTPases J Biol Chem 2007 282 35666 35678 10.1074/jbc.M703571200 17932039

[B37] Stenmark H Rab GTPases as coordinators of vesicle traffic Nat Rev Mol Cell Biol 2009 10 513 525 19603039 10.1038/nrm2728

[B38] Brown TC Tran IC Backos DS Esteban JA NMDA receptor-dependent activation of the small GTPase Rab5 drives the removal of synaptic AMPA receptors during hippocampal LTD Neuron 2005 45 81 94 10.1016/j.neuron.2004.12.023 15629704

[B39] Sun J Deghmane AE Bucci C Hmama Z Detection of activated Rab7 GTPase with an immobilized RILP probe Methods Mol Biol 2009 531 57 69 10.1007/978-1-59745-396-7_5 19347311

[B40] Roberts RL Barbieri MA Pryse KM Chua M Morisaki JH Stahl PD Endosome fusion in living cells overexpressing GFP-rab5 J Cell Sci 1999 112 3667 3675 10523503 10.1242/jcs.112.21.3667

[B41] Kruth HS Jones NL Huang W Zhao B Ishii I Chang J Combs CA Malide D Zhang WY Macropinocytosis is the endocytic pathway that mediates macrophage foam cell formation with native low density lipoprotein J Biol Chem 2005 280 2352 2360 15533943 10.1074/jbc.M407167200

[B42] Manabe T Yoshimori T Henomatsu N Tashiro Y Inhibitors of vacuolar-type H(+)-ATPase suppresses proliferation of cultured cells J Cell Physiol 1993 157 445 452 10.1002/jcp.1041570303 8253855

[B43] Lefranc F Brotchi J Kiss R Possible future issues in the treatment of glioblastomas: special emphasis on cell migration and the resistance of migrating glioblastoma cells to apoptosis J Clin Oncol 2005 23 2411 2422 10.1200/JCO.2005.03.089 15800333

[B44] Ishii N Maier D Merlo A Tada M Sawamura Y Diserens A-C Van Meir EG Frequnt co-alterations of TP53, p16/CDKN2A, p14arf, PTEN tumor suppressor genes in human glioma cell lines Brain Pathol 1999 9 469 479 10416987 10.1111/j.1750-3639.1999.tb00536.x PMC8098486

[B45] Furnari FB Fenton T Bachoo RM Mukasa A Stommel JM Stegh A Hahn WC Ligon KL Louis DN Brennan C Chin L DePinho RA Cavenee WK Malignant astrocytic glioma: genetics, biology, and paths to treatment Genes Dev 2007 21 2683 2710 10.1101/gad.1596707 17974913

[B46] Stupp R Mason WP van den Bent MJ Weller M Fisher B Taphoorn MJ Belanger K Brandes AA Marosi C Bogdahn U Curschmann J Janzer RC Ludwin SK Gorlia T Allgeier A Lacombe D Cairncross JG Eisenhauer E Mirimanoff RO Radiotherapy plus concomitant and adjuvant temozolomide for glioblastoma N Engl J Med 2005 352 987 996 10.1056/NEJMoa043330 15758009

[B47] Gunther W Pawlak E Damasceno R Arnold H Terzis AJ Temozolomide induces apoptosis and senescence in glioma cells cultured as multicellular spheroids Br J Cancer 2003 88 463 469 10.1038/sj.bjc.6600711 12569392 PMC2747547

[B48] Roos WP Batista LF Naumann SC Wick W Weller M Menck CF Kaina B Apoptosis in malignant glioma cells triggered by the temozolomide-induced DNA lesion O6-methylguanine Oncogene 2007 26 186 197 10.1038/sj.onc.1209785 16819506

[B49] Kanzawa T Germano IM Komata T Ito H Kondo Y Kondo S Role of autophagy in temozolomide-induced cytotoxicity for malignant glioma cells Cell Death Differ 2004 11 448 457 10.1038/sj.cdd.4401359 14713959

[B50] Lefranc F Facchini V Kiss R Proautophagic drugs: a novel means to combat apoptosis-resistant cancers, with a special emphasis on glioblastomas Oncologist 2007 12 1395 1403 10.1634/theoncologist.12-12-1395 18165616

[B51] Yip S Miao J Cahill DP Iafrate AJ Aldape K Nutt CL Louis DN MSH6 mutations arise in glioblastomas during temozolomide therapy and mediate temozolomide resistance Clin Cancer Res 2009 15 4622 4629 10.1158/1078-0432.CCR-08-3012 19584161 PMC2737355

[B52] Zhang J Stevens MF Laughton CA Madhusudan S Bradshaw TD Acquired resistance to temozolomide in glioma cell lines: molecular mechanisms and potential translational applications Oncology 2010 78 103 114 10.1159/000306139 20357518

[B53] Lefranc F Kiss R The sodium pump alpha1 subunit as a potential target to combat apoptosis-resistant glioblastomas Neoplasia 2008 10 198 206 18323016 10.1593/neo.07928 PMC2259449

[B54] Porat-Shliom N Kloog Y Donaldson JG A unique platform for H-Ras signaling involving clathrin-independent endocytosis Mol Biol Cell 2008 19 765 775 18094044 10.1091/mbc.E07-08-0841 PMC2262976

[B55] Donaldson JG Porat-Shliom N Cohen LA Clathrin-independent endocytosis: a unique platform for cell signaling and PM remodeling Cell Signal 2009 21 1 6 10.1016/j.cellsig.2008.06.020 18647649 PMC2754696

[B56] Schnatwinkel C Christoforidis S Lindsay MR Uttenweiler-Joseph S Wilm M Parton RG Zerial M The Rab5 effector Rabankyrin-5 regulates and coordinates different endocytic mechanisms PLoS Biol 2004 2 e261 10.1371/journal.pbio.0020261 15328530 PMC514490

[B57] Kerr MC Lindsay MR Luetterforst R Hamilton N Simpson F Parton RG Gleeson PA Teasdale RD Visualisation of macropinosome maturation by the recruitment of sorting nexins J Cell Sci 2006 119 3967 3980 10.1242/jcs.03167 16968745

[B58] Racoosin EL Swanson JA Macropinosome maturation and fusion with tubular lysosomes in macrophages J Cell Biol 1993 121 1011 1020 10.1083/jcb.121.5.1011 8099075 PMC2119679

[B59] Gold S Monaghan P Mertens P Jackson T A clathrin independent macropinocytosis-like entry mechanism used by bluetongue virus-1 during infection of BHK cells PLoS One 2010 5 e11360 10.1371/journal.pone.0011360 20613878 PMC2894058

[B60] Luzio JP Parkinson MD Gray SR Bright NA The delivery of endocytosed cargo to lysosomes Biochem Soc Trans 2009 37 1019 1021 10.1042/BST0371019 19754443

[B61] Wang T Ming Z Xiaochun W Hong W Rab7: Role of its protein interaction cascades in endo-lysosomal traffic Cell Signal 2011 23 516 521 10.1016/j.cellsig.2010.09.012 20851765

[B62] Le Calve B Rynkowski M Le Mercier M Bruyere C Lonez C Gras T Haibe-Kains B Bontempi G Decaestecker C Ruysschaert JM Kiss R Lefranc F Long-term in vitro treatment of human glioblastoma cells with temozolomide increases resistance in vivo through up-regulation of GLUT transporter and aldo-keto reductase enzyme AKR1C expression Neoplasia 2010 12 727 739 20824049 10.1593/neo.10526 PMC2933693

[B63] Medina OP Zhu Y Kairemo K Targeted liposomal drug delivery in cancer Curr Pharm Des 2004 10 2981 2989 10.2174/1381612043383467 15379663

[B64] Praetorius NP Mandal TK Engineered nanoparticles in cancer therapy Recent Pat Drug Deliv Formul 2007 1 37 51 10.2174/187221107779814104 19075873

[B65] Passarella RJ Spratt DE van der Ende AE Phillips JG Wu H Sathiyakumar V Zhou L Hallahan DE Harth E Diaz R Targeted nanoparticles that deliver a sustained, specific release of Paclitaxel to irradiated tumors Cancer Res 2010 70 4550 4559 10.1158/0008-5472.CAN-10-0339 20484031 PMC2880200

[B66] Maltese WA De Vivo DC Cholesterol and phospholipids in cultured skin fibroblasts from patients with dystonia Ann Neurol 1984 16 250 252 10.1002/ana.410160215 6476796

[B67] Debnath J Muthuswamy SK Brugge JS Morphogenesis and oncogenesis of MCF-10A mammary epithelial acini grown in three-dimensional basement membrane cultures Methods 2003 30 256 268 10.1016/S1046-2023(03)00032-X 12798140

[B68] Kaul A Overmeyer JH Maltese WA Activated Ras induces cytoplasmic vacuolation and non-apoptotic death in glioblastoma cells via novel effector pathways Cell Signal 2007 19 1034 1043 10.1016/j.cellsig.2006.11.010 17210246 PMC1894854

[B69] Romero RK Peralta ER Guenther GG Wong SY Edinger AL Rab7 activation by growth factor withdrawal contributes to the induction of apoptosis Mol Biol Cell 2009 20 2831 2840 10.1091/mbc.E08-09-0911 19386765 PMC2695791

[B70] Johnson EE Overmeyer JH Gunning WT Maltese WA Gene silencing reveals a specific function of hVps34 phosphatidylinositol 3-kinase in late versus early endosomes J Cell Sci 2006 119 1219 1232 10.1242/jcs.02833 16522686

[B71] Zeng X Overmeyer JH Maltese WA Functional specificity of the mammalian Beclin-Vps34 PI 3-kinase complex in macroautophagy versus endocytosis and lysosomal enzyme trafficking J Cell Sci 2006 119 259 270 10.1242/jcs.02735 16390869

