# Supplementary figures and images for: A chalcone-related small molecule that induces methuosis, a novel form of non-apoptotic cell death, in glioblastoma cells
Source: Mol Cancer. 2011 Jun 6;10:69. doi: 10.1186/1476-4598-10-69 (PMC3118192; doi:10.1186/1476-4598-10-69)

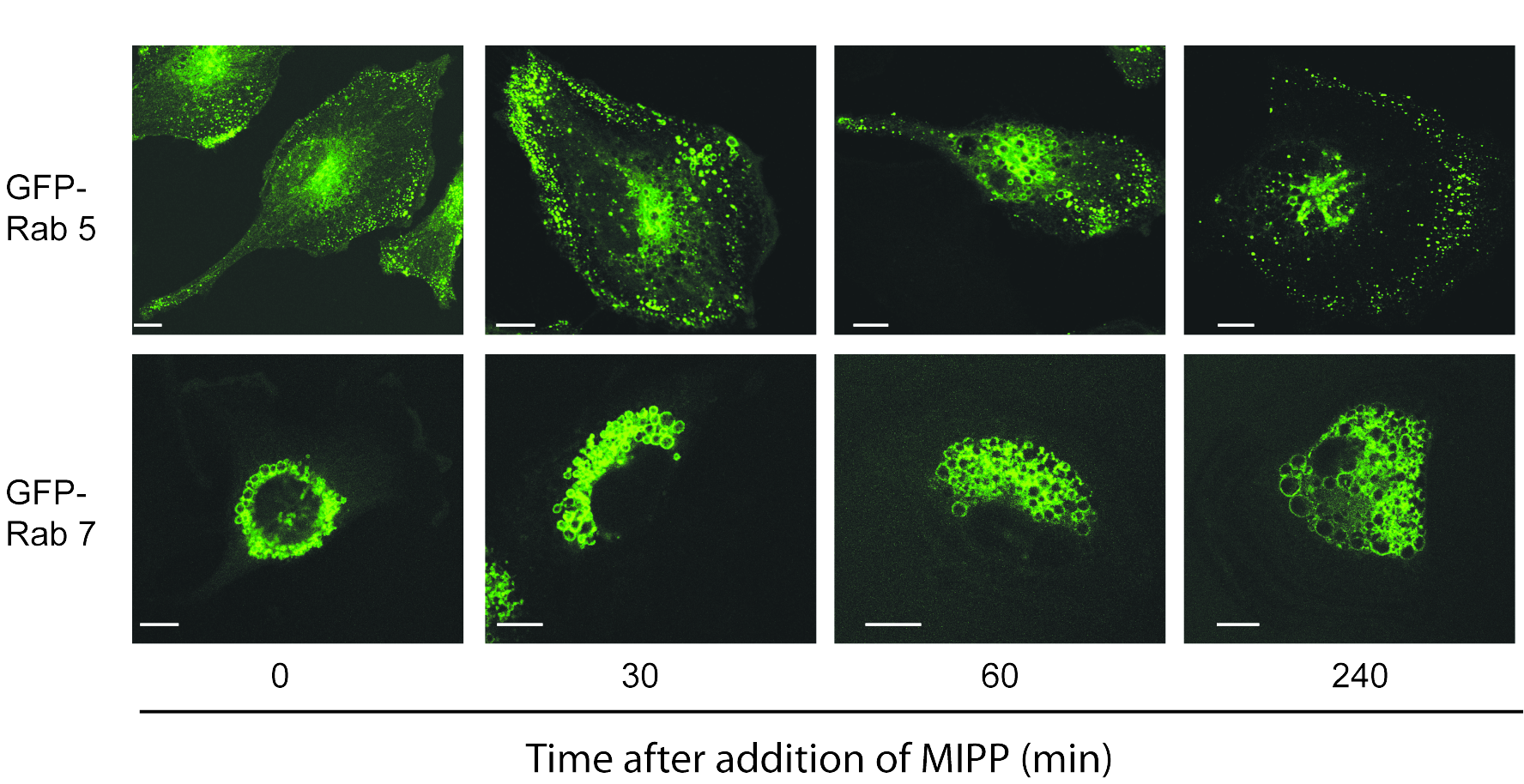

Supplement: Additional file 3 — Figure S1. U251 cells were transfected with expression vectors encoding GFP-Rab5 or GFP-Rab7. After 24 h, MIPP was added at a concentration of 10 μM and cells were examined by confocal fluorescence microscopy at the indicated intervals. The results show that even at the earliest time points most of the vacuoles are decorated with Rab7. In contrast, Rab5 is mostly localized in smaller punctate structures. The scale bars are 10 microns. [file 1476-4598-10-69-S3.TIFF]

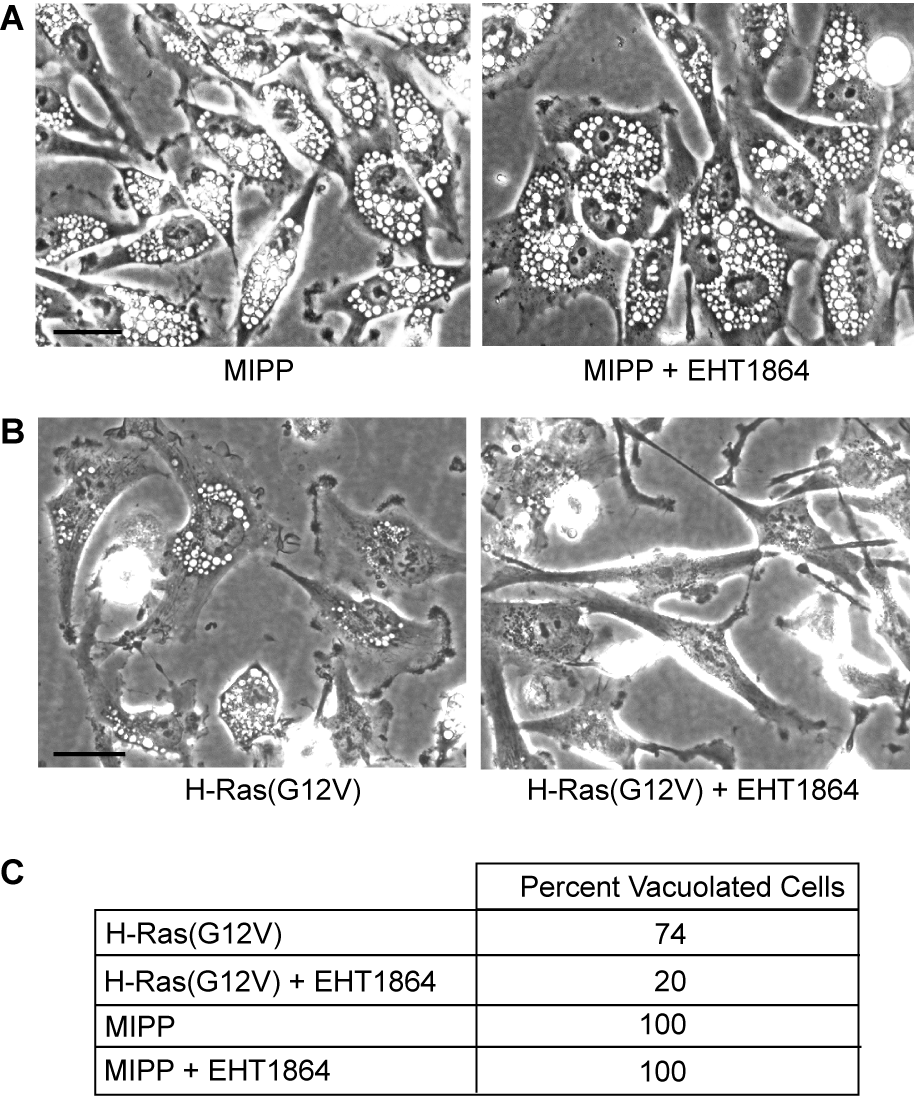

Supplement: Additional file 4 — Figure S2. The Rac inhibitor, EHT 1864, does not block the induction of vacuoles by MIPP. U251 cells were incubated with MIPP for 24 h in the presence or absence of 25 μM EHT 1864 (panel A). In a separate experiment, U251 cells were incubated with or without EHT 1864 following nucleofection with a vector encoding a constitutively active H-Ras(G12V) (panel B). Phase-contrast images were taken 24 h after addition of the Rac inhibitor. The scale bars are 10 microns. C) The percentage of vacuolated cells for each condition was determined as described in Materials and Methods. [file 1476-4598-10-69-S4.TIFF]

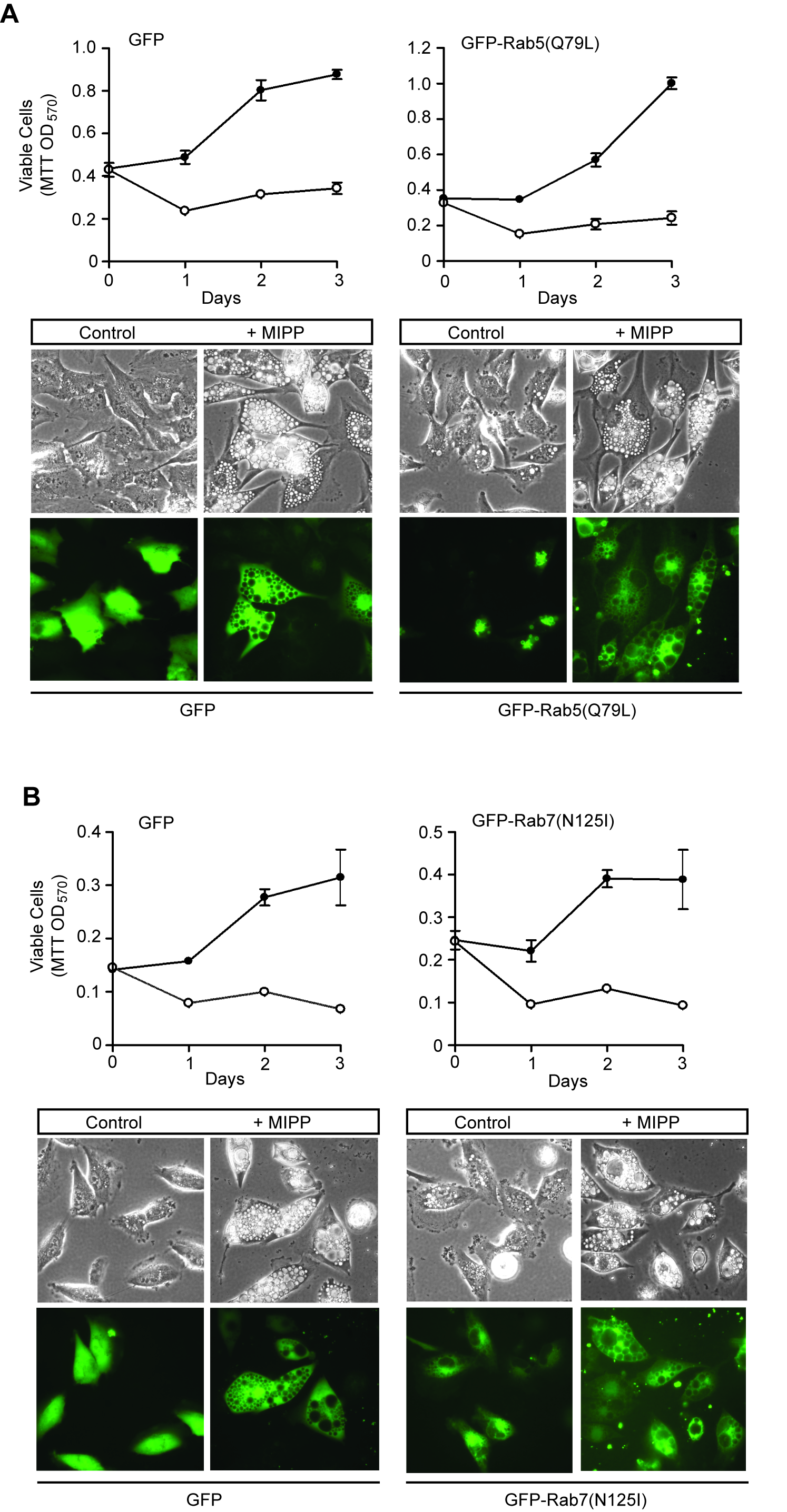

Supplement: Additional file 5 — Figure S3. Expression of constitutively active Rab5 or dominant-negative Rab7 does not protect U251 cells from MIPP-induced methuosis. A) Cells were nucleofected with vectors encoding GFP or constitutively active GFP-Rab5(Q79L), as indicated above each panel. One day after nucleofection (time-0) parallel cultures were treated with 10 μM MIPP (○) or an equivalent volume of DMSO (●) and viable cells were measured by MTT assay. Cells in parallel dishes were subjected to fluorescence and phase contrast microcopy to evaluate the extent to which the nucleofected cells (green) were vacuolated. B) The identical experiment was conducted with cells expressing GFP or the dominant-negative GFP-Rab7(N125I). [file 1476-4598-10-69-S5.TIFF]

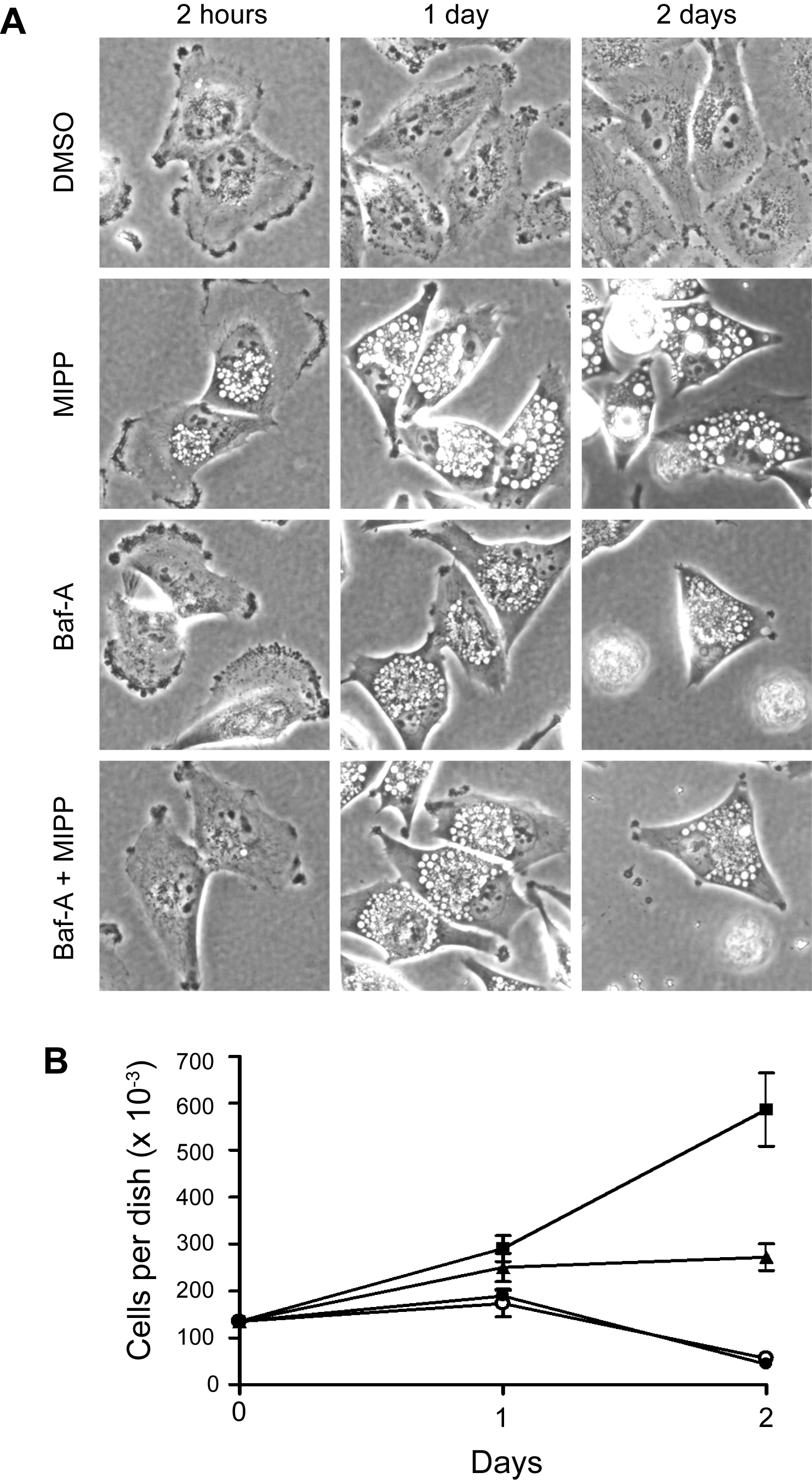

Supplement: Additional file 6 — Figure S4. Long-term treatment of U251 cells with Bafilomycin A1 (Baf-A) induces vacuolization and is cytotoxic. Therefore, Baf-A cannot protect cells from MIPP-induced methuosis. U251 cells were treated for 2 days with the indicated compounds. A) Cells were examined by phase contrast microscopy to assess vacuolization. B) Cell growth was assessed by counting attached cells in 3 parallel cultures (mean ± SD) at each time point. The symbols are: DMSO control (■), 10 μM MIPP alone (▲), 50 nM Baf-A alone (○), or a combination of 10 μM MIPP plus 50 nM Baf-A (●). [file 1476-4598-10-69-S6.TIFF]

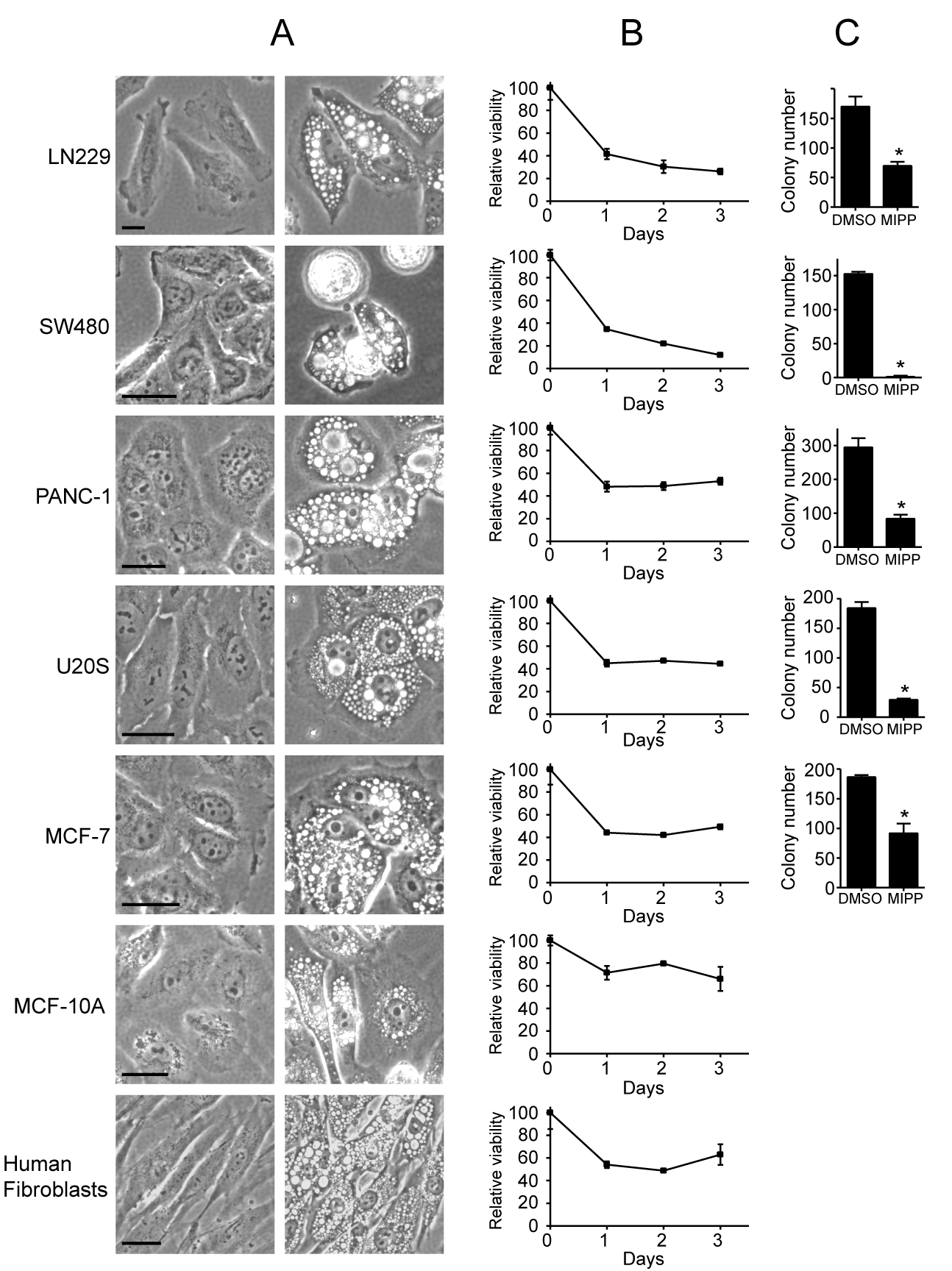

Supplement: Additional file 7 — Figure S5. MIPP induces vacuoles and inhibits growth and viability in multiple human cell lines. Cell lines examined were: LN229, glioblastoma; SW480, colon adenocarcinoma; PANC-1, pancreatic carcinoma; U20S, osteosarcoma; MCF-7, mammary adenocarcinoma; MCF-10A, mammary epithelial cells; primary human skin fibroblasts. A) Phase-contrast images of cells were acquired after two days of treatment with 10 μM MIPP. The scale bars are 10 microns. B) MTT assays were performed on cells treated for the indicated number of days with 10 μM MIPP or an equivalent volume of DMSO. The MTT values for the MIPP-treated wells were expressed as percent of the mean for the parallel DMSO-treated wells. Error bars indicate the SD. C) Colony-forming assays for the transformed cell lines were preformed as described in Materials and Methods. Values are the mean (± SD) from triplicate cultures. All of the decreases in colony formation (*) were significant at p < 0.001. [file 1476-4598-10-69-S7.TIFF]
